# Next‐generation transgenic cotton: pyramiding RNAi and Bt counters insect resistance

**DOI:** 10.1111/pbi.12709

**Published:** 2017-03-16

**Authors:** Mi Ni, Wei Ma, Xiaofang Wang, Meijing Gao, Yan Dai, Xiaoli Wei, Lei Zhang, Yonggang Peng, Shuyuan Chen, Lingyun Ding, Yue Tian, Jie Li, Haiping Wang, Xiaolin Wang, Guowang Xu, Wangzhen Guo, Yihua Yang, Yidong Wu, Shannon Heuberger, Bruce E. Tabashnik, Tianzhen Zhang, Zhen Zhu

**Affiliations:** ^1^ State Key Laboratory of Plant Genomics and National Center for Plant Gene Research (Beijing) Institute of Genetics and Developmental Biology Chinese Academy of Sciences Beijing China; ^2^ National Key Laboratory for Crop Genetics and Germplasm Enhancement Jiangsu Plant Gene Engineering Research Center Nanjing Agricultural University Nanjing China; ^3^ College of Plant Protection Nanjing Agricultural University Nanjing China; ^4^ Key Laboratory of Separation Science for Analytical Chemistry Dalian Institute of Chemical Physics Chinese Academy of Sciences Dalian China; ^5^ Department of Entomology University of Arizona Tucson AZ USA

**Keywords:** genetic engineering, RNA interference, *Bacillus thuringiensis*, juvenile hormone, *Helicoverpa armigera*, sustainability

## Abstract

Transgenic crops producing insecticidal proteins from the bacterium *Bacillus thuringiensis* (Bt) are extensively cultivated worldwide. To counter rapidly increasing pest resistance to crops that produce single Bt toxins, transgenic plant ‘pyramids’ producing two or more Bt toxins that kill the same pest have been widely adopted. However, cross‐resistance and antagonism between Bt toxins limit the sustainability of this approach. Here we describe development and testing of the first pyramids of cotton combining protection from a Bt toxin and RNA interference (RNAi). We developed two types of transgenic cotton plants producing double‐stranded RNA (dsRNA) from the global lepidopteran pest *Helicoverpa armigera* designed to interfere with its metabolism of juvenile hormone (JH). We focused on suppression of JH acid methyltransferase (JHAMT), which is crucial for JH synthesis, and JH‐binding protein (JHBP), which transports JH to organs. In 2015 and 2016, we tested larvae from a Bt‐resistant strain and a related susceptible strain of *H. armigera* on seven types of cotton: two controls, Bt cotton, two types of RNAi cotton (targeting JHAMT or JHBP) and two pyramids (Bt cotton plus each type of RNAi). Both types of RNAi cotton were effective against Bt‐resistant insects. Bt cotton and RNAi acted independently against the susceptible strain. In computer simulations of conditions in northern China, where millions of farmers grow Bt cotton as well as abundant non‐transgenic host plants of *H. armigera*, pyramided cotton combining a Bt toxin and RNAi substantially delayed resistance relative to using Bt cotton alone.

## Introduction

Transgenic crops producing *Bacillus thuringiensis* (Bt) toxins are used widely for insect control, with more than 84 million hectares of Bt cotton, corn and soybean planted globally in 2015 (James, 2016). These Bt crops can suppress pests, reduce insecticide use and increase farmer profits (Hutchison *et al*., 2010; Lu *et al*., 2012; NASEM, 2016; Tabashnik *et al*., 2010; Wu *et al*., 2008a). Because some pests have rapidly evolved resistance to transgenic crops that make only one Bt toxin, farmers have been switching to Bt crop ‘pyramids’ producing two or more toxins that kill the same pest (Carrière *et al*., 2015; Farias *et al*., 2014; Gassmann *et al*., 2014; Jakka *et al*., 2016; Tabashnik *et al*., 2013; Wu, 2014; Zhang *et al*., 2012; Zukoff *et al*., 2016). However, the efficacy and durability of such pyramids are reduced by cross‐resistance and antagonism between Bt toxins (Carrière *et al*., 2015, 2016), so alternative management tactics are urgently needed (Tabashnik, 2016).

Either as an alternative or a complement to Bt toxins, RNA interference (RNAi) has great promise for insect pest control (Asokan *et al*., 2014; Baum and Roberts, 2014; Baum *et al*., 2007; Fishilevich *et al*., 2016; Fu *et al*., 2016; Kim *et al*., 2015; Levine *et al*., 2015; Lim *et al*., 2016; Mao *et al*., 2007; Tian *et al*., 2015; Yu *et al*., 2016). With RNAi, small double‐stranded RNA (dsRNA) causes sequence‐specific suppression of target gene expression. To achieve safe and effective pest control with RNAi, the goal is to reduce expression of genes encoding proteins that are essential to pests, but not to other organisms. For example, potential targets for RNAi include genes encoding proteins that synthesize or transport juvenile hormones (JHs) (Asokan *et al*., 2014; Fu *et al*., 2016; Tian *et al*., 2015), which are critical for insect development, yet absent from most other organisms (Belles *et al*., 2005). Although corn protected against some beetles by a pyramid of Bt toxins and RNAi is under development (Baum and Roberts, 2014; Fishilevich *et al*., 2016; Levine *et al*., 2015), previous papers have not reported the efficacy of Bt plus RNAi pyramids against Bt‐resistant or susceptible strains of target pests. Also, despite recognition of the threat of field‐evolved pest resistance to RNAi (Baum and Roberts, 2014; Fishilevich *et al*., 2016), previous work has not used computer modelling to systematically assess this risk.

Here we created transgenic cotton pyramids that combine protection from Bt and RNAi against *Helicoverpa armigera*, one of the world's most destructive pests of cotton and many other crops (Tay *et al*., 2013). We focused on suppression of JH acid methyltransferase (JHAMT), which is crucial for a final step of JH synthesis (Minakuchi *et al*., 2008; Shinoda and Itoyama, 2003), and JH‐binding protein (JHBP), which transports JH from the haemolymph to the cells of target organs (Pietrzyk *et al*., 2013; Suzuki *et al*., 2011). In previous work with *H. armigera*, dsRNA aimed at JHAMT greatly reduced pupation (Asokan *et al*., 2014). Here we tested larvae from a Bt‐resistant strain (SCD‐r1) and a related susceptible strain (SCD) of *H. armigera* in 2015 and 2016 on seven types of cotton plants: two controls, Bt cotton, two types of RNAi cotton (targeting JHAMT or JHBP) and two pyramids (Bt cotton plus each type of RNAi). In all plants in this study, the Bt toxin gene was derived from cotton variety GK19, which has been used widely in China and produces a Cry1Ac/Cry1Ab chimeric protein that is 98.5% identical to Cry1Ac (Guo *et al*., 1995; Tabashnik *et al*., 2012; Wan *et al*., 2005; Zhang *et al*., 1998). Computer modelling incorporating the data from bioassays shows that the pyramids can substantially delay evolution of resistance, especially under conditions simulating northern China, where large refuges of non‐transgenic host plants are available (Jin *et al*., 2015).

## Results

### Genes encoding JHAMT and JHBP proteins in *H. armigera*

We amplified and cloned from third instar larvae of *H. armigera* the genes predicted to encode JHAMT (*HaJHAMT*) and JHBP (*HaJHBP*) **(**GenBank accession nos. KX289532 and KX289533, respectively, Figures S1–S4). The *HaJHAMT* cDNA encodes a predicted protein (HaJHAMT) of 284 amino acids that contains conserved S‐adenosyl‐L‐methionine (SAM)‐binding motif I (Kagan and Clarke, 1994) (Figures S1 and S2) and has identity of 98% to HaJHAMT predicted from a Japanese strain (AB127945) and 100% identity to a partial sequence (259 amino acids) of HaJHAMT predicted from an Indian strain (GU323798). The *HaJHBP* cDNA encodes a predicted protein (HaJHBP) of 242 amino acids that contains conserved elements involved in JH recognition and binding (Wojtasek and Prestwich, 1995) (Figures S3 and S4) and has 98% identity to HaJHBP predicted from independent sequencing in a different laboratory in China (KT443972). Transcription of *HaJHAMT* and *HaJHBP* in *H. armigera* larvae peaked in fourth instars, in which it occurred primarily in the head for *HaJHAMT* and in the fat body for *HaJHBP* (Figures S5 and S6).

### Efficacy of JH dsRNAs in artificial diet against *H. armigera* larvae

**Figure 1:**
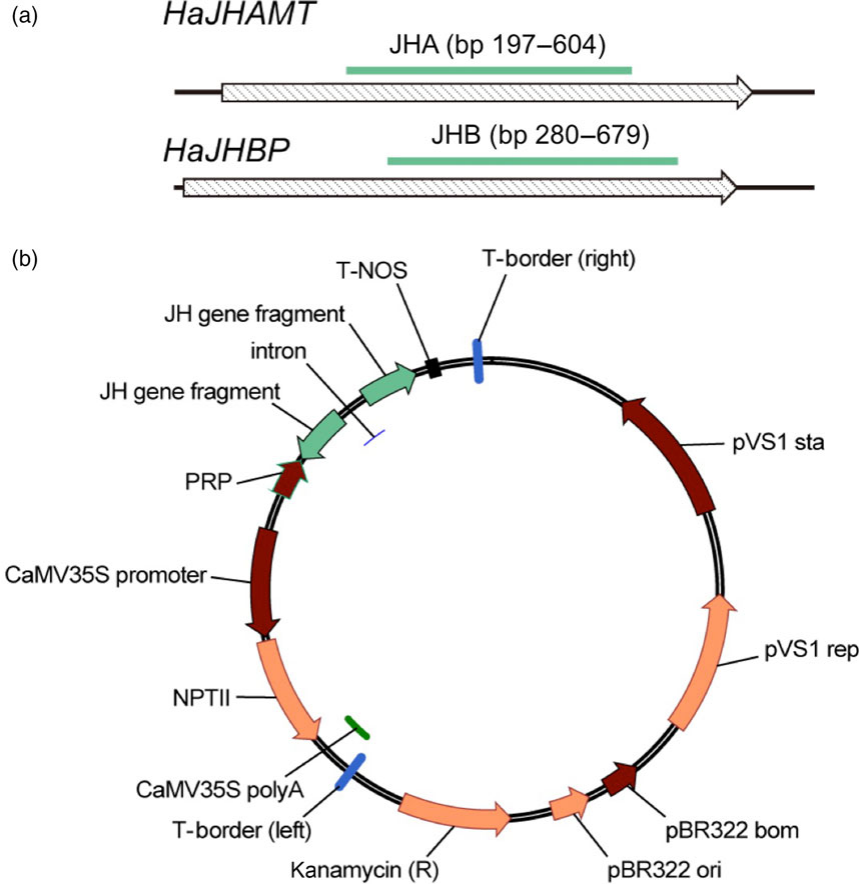
Fragments of *H. armigera* genes and vectors used to transform cotton for RNAi affecting juvenile hormone (JH). (a) Gene fragments JHA from *HaJHAMT* and JHB from *HaJHBP* (see Figures S1–S4 for sequences). (b) Vectors used for *Agrobacterium*‐mediated transformation of cotton. We modified plasmid pCAMBIA2300 so dsRNA expression was controlled by promoter PRP from cotton leaf curl virus upstream from fragment JHA, JHB or GFP (negative control) in the sense orientation, an intron from the potato GA20‐oxidase gene, the same fragment in the antisense orientation and a nopaline synthase (NOS) terminator (see Experimental Procedures).

We targeted two *H. armigera* DNA sequences: JHA (408 bp) and JHB (400 bp), which code for portions of HaJHAMT and HaJHBP, respectively (Figures 1a and S1–S4). Excluding moths in the same family as *H. armigera* such as *Spodoptera litura*, these two sequences lack 21 bp exact matches to homologous genes in several other insect species including the silkworm, *Bombyx mori* (Figures S1 and S3). In artificial diet bioassays with *H. armigera* larvae, treatment with dsRNA from JHA or JHB significantly increased mortality and decreased weight relative to a control treatment with dsRNA from green fluorescent protein (GFP) (Figure S7).

### Transgenic cotton producing dsRNA to disrupt JH synthesis and transport

We constructed vectors expressing dsRNA from JHA, JHB or green fluorescent protein (GFP) (Figure 1b) and used *Agrobacterium*‐mediated transformation (Figure S8) with a binary vector system to generate pure lines of transgenic cotton with a single insert of JHA, JHB or GFP, referred to hereafter as JHA, JHB or GFP cotton, respectively.

**Figure 2:**
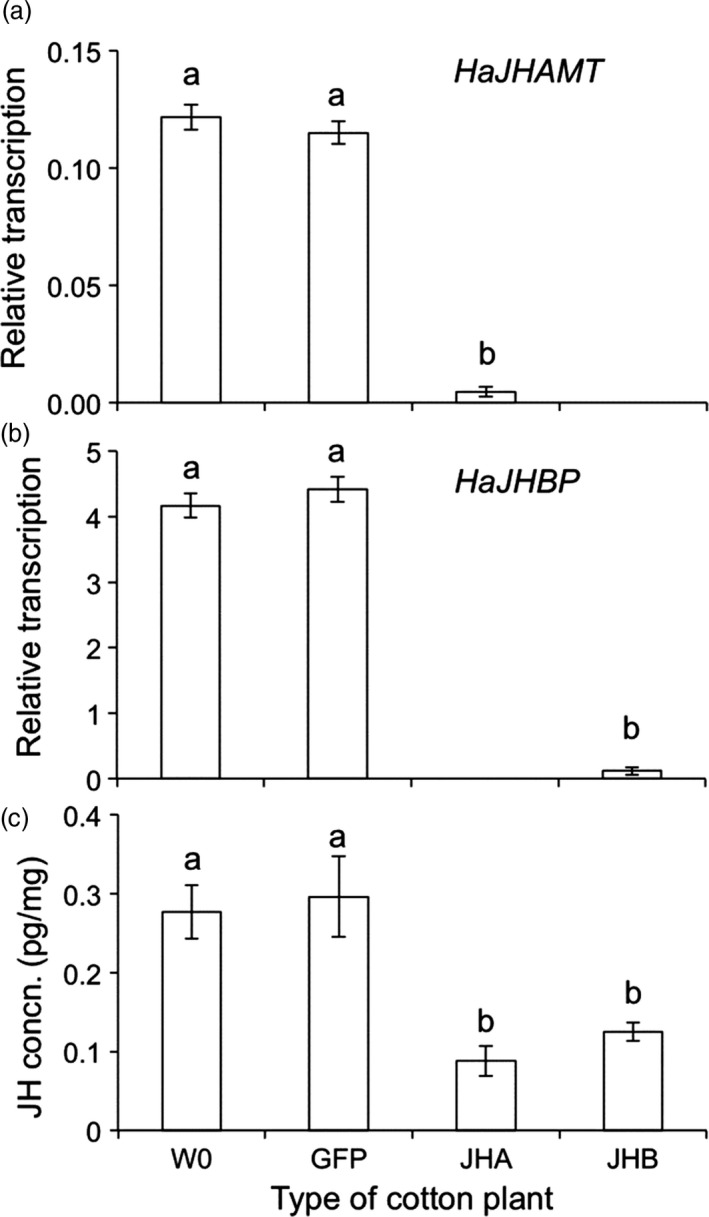
Effects on transcription and JH concentration of feeding by susceptible (SCD) larvae of *H. armigera* on leaves from four types of cotton plants: non‐transgenic parent (W0), transgenic control (GFP), transgenic cotton producing dsRNA from *HaJHAMT* (JHA) or *HaJHBP* (JHB). (a) Transcription of *HaJHAMT* relative to actin. (b) Transcription of *HaJHBP* relative to actin. Larvae were not fed JHB cotton in (a) or JHA cotton in (b). Relative transcription was significantly lower for either JHA or JHB cotton than for W0 or GFP (*P* = 0.001 in each case, Tukey's HSD). (c) JH concentration was significantly lower for JHA and JHB than either W0 or GFP (*P* < 0.05 in each case, Tukey's HSD). No significant difference occurred between W0 and GFP in relative transcription or JH concentration (*P* > 0.5 in each case, Tukey's HSD). Bars show means and SE with an average of 4.6 replicates per bar.

The initial transformation of kanamycin‐resistant plants was verified by PCR detection of the transgenic dsRNA expression cassette (Figure S9). The positive lines were selfed to generate homozygous second‐generation (T2) progeny. We used Southern analysis to detect lines in which the third‐generation (T3) progeny contained a single copy of the transgene insertion (Figures S10 and S11). These lines were used in subsequent experiments. The three transgenic cotton lines created here (JHA, JHB and GFP cotton) were made by introducing transgenes into the same conventional variety of cotton (W0) and thus share a common genetic background. As expected, feeding by susceptible (SCD) larvae of *H. armigera* on leaves of JHA and JHB cotton suppressed transcription of *HaJHAMT* and *HaJHBP*, respectively, and reduced JH concentration (Figure 2).

### Transgenic cotton pyramids: Bt + RNAi

To generate pyramided cotton protected by a Bt toxin and RNAi, we crossed Bt cotton variety GK19 with either JHA or JHB cotton. The first‐generation (F1) offspring from these crosses were heterozygous for both traits, and these pyramided plants were used for bioassays in 2015 (see below). To generate cotton plants homozygous for both traits, we crossed the F1 plants by self‐pollination to produce F2 offspring. We used real‐time PCR to determine which F2 plants had the same copy number of both the Bt gene and either JHA or JHB as the pure, parental transgenic lines for each trait. As expected, approximately 6% (1/16) of the F2 offspring met this criterion. We used the homozygous pyramided plants for bioassays in 2016 (see below).

### Efficacy of Bt cotton, RNAi cotton and Bt + RNAi cotton against resistant and susceptible *H. armigera*

**Figure 3:**
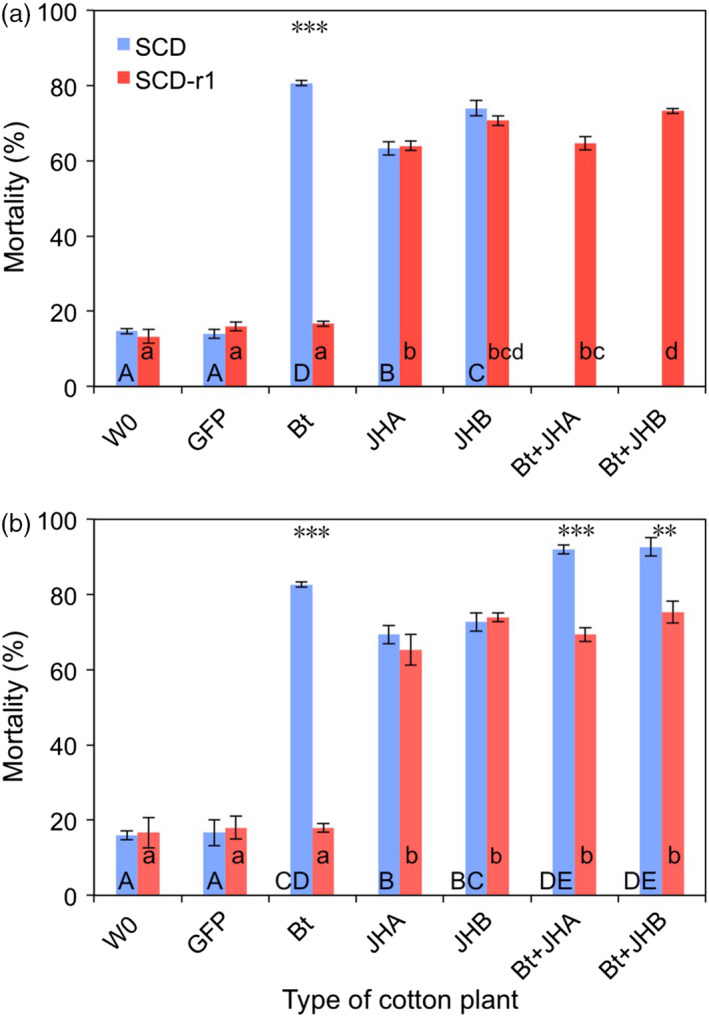
Mortality of susceptible (SCD) and resistant (SCD‐r1) larvae of *H. armigera* fed leaves from seven types of cotton plants: non‐transgenic parent (W0), transgenic control (GFP), *B. thuringiensis* (Bt), RNAi (JHA and JHB) and pyramids (Bt + JHA and Bt + JHB). (a) 2015. (b) 2016. Each bar shows the mean and SE for mortality (%) based on three replicates of 50 larvae per replicate (*n* = 150 per bar, total *n* = 3900). For a given type of cotton and year, asterisks indicate a significant difference between insect strains (*t*‐tests, ***: *P* < 0.001, **: *P* = 0.01, no asterisks: NS, *P* > 0.20). For a given insect strain and year, different letters indicate significant differences between types of cotton; upper case for SCD and lower case for SCD‐r1 (Tukey's HSD, *P* < 0.05). SCD was not tested on pyramids in 2015.

**Figure 4:**
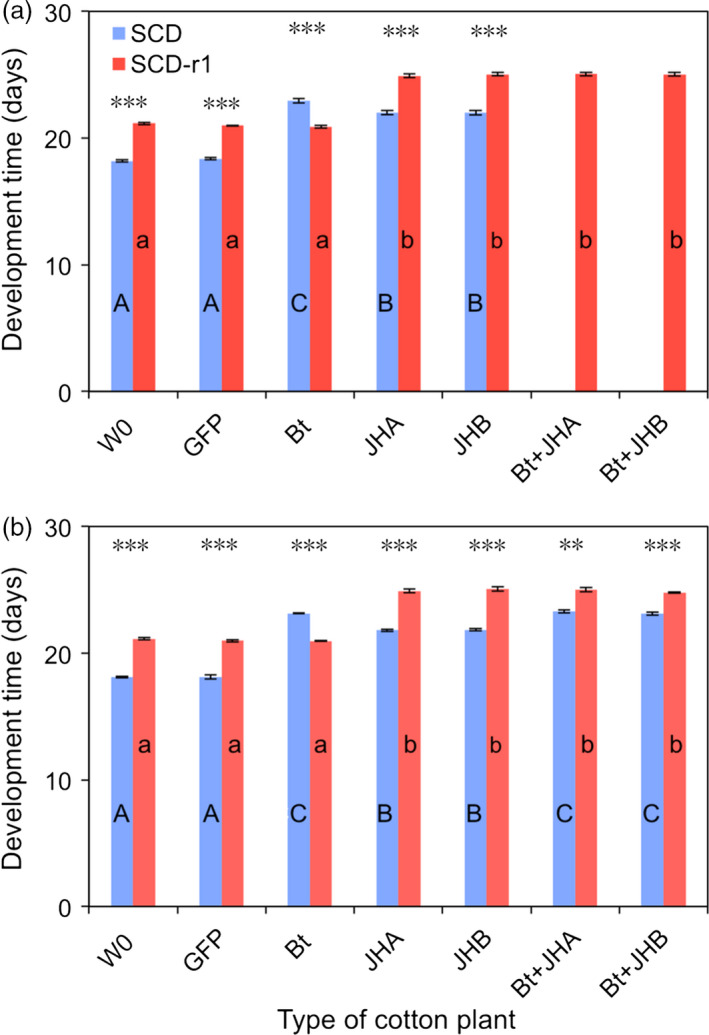
Development time for susceptible (SCD) and resistant (SCD‐r1) larvae of *H. armigera* fed leaves from seven types of cotton plants: non‐transgenic parent (W0), transgenic control (GFP), *B. thuringiensis* (Bt), RNAi (JHA and JHB) and pyramids (Bt + JHA and Bt + JHB). (a) 2015. (b) 2016. Each bar shows the mean and SE for days from neonate to pupa based on three replicates (mean sample size per bar = 72 pupae, total *n *= 1884 pupae). For a given type of cotton and year, asterisks indicate a significant difference between insect strains (*t*‐tests, ***: *P* < 0.001, **: *P* = 0.0012). For a given insect strain and year, different letters indicate significant differences between types of cotton; upper case for SCD and lower case for SCD‐r1 (Tukey's HSD, *P* < 0.05). SCD was not tested on pyramids in 2015.

Results from bioassays with cotton leaves in 2015 and 2016 confirm that the Cry1Ac‐resistant strain (SCD‐r1) was highly resistant to Bt cotton (Figures 3 and 4). For SCD‐r1, mortality (%) and development time (days to pupation) did not differ significantly between Bt cotton and either the conventional cotton (W0) or the transgenic control cotton (GFP) (Figures 3 and 4). In contrast, for the susceptible strain SCD, mortality and development time were significantly greater on Bt cotton than on both of the control cottons (W0 and GFP) (Figures 3 and 4). We calculated the efficacy (%) of transgenic cotton as 100% minus the survival (%) on transgenic cotton divided by the survival (%) on non‐transgenic cotton (W0). The efficacy of Bt cotton was 77.4%–79.4% against SCD and only 1.6%–3.9% against SCD‐r1 (Figure 3).

Despite its strong resistance to Bt cotton, SCD‐r1 had no cross‐resistance to either JHA or JHB cotton (Figures 3 and 4). On JHA and JHB cotton, mortality did not differ significantly between SCD‐r1 and SCD (Figure 3) and development time was significantly greater for SCD‐r1 than SCD (Figure 4). For each type of RNAi cotton, efficacy was similar against SCD and SCD‐r1 (Figure 3). The efficacy of JHA cotton was 57.0%–63.5% against SCD and 58.4%–58.5% against SCD‐r1 (Figure 3). The efficacy of JHB cotton was 67.5%–69.5% against SCD and 66.2%–68.8% against SCD‐r1 (Figure 3).

For SCD‐r1, mortality and development time did not differ significantly between the pyramids of Bt + RNAi cotton and each of the corresponding RNAi cottons without Bt toxin (Figures 3 and 4). Consistent with the results summarized above, these data indicate that the Bt toxin in the pyramids did not affect the mortality or development time of SCD‐r1.

By contrast, against SCD, both pyramids were more effective than the RNAi cottons (Figures 3 and 4). For SCD, mortality was significantly greater on the pyramids (92.0% for Bt + JHA and 92.7% for Bt + JHB) than on the RNAi cottons (69.3% for JHA and 72.7% for JHB) (Figure 3). Likewise, development time (days to pupation) for SCD was significantly greater on the pyramids (23.2 for Bt + JHA and 23.1 for Bt + JHB) than on the RNAi cottons (21.8 for both JHA and JHB) (Figure 3). The efficacy of Bt + JHA cotton was 90.5% against SCD and 59.3%–69.2% against SCD‐r1 (Figure 4). The efficacy of Bt + JHB cotton was 91.3% against SCD and 69.2%–70.4% against SCD‐r1 (Figure 4).

To determine whether the Bt toxin and RNAi in the pyramids act independently against SCD, we calculated the index of multiplicative survival (IMS), which is the observed survival on a two‐trait pyramid (i.e. Bt + RNAi) divided by the survival expected on the pyramid based on multiplying the survival on each type of the single‐trait plants (Carrière *et al*., 2015). An IMS value of 1 indicates independent action of the two traits in the pyramid (Carrière *et al*., 2015). The mean IMS value was 1.3 for Bt + JHA (SE = 0.18) and 1.3 for Bt + JHB (SE = 0.43), which does not differ significantly from 1 (one‐sample *t*‐test for pooled data, *t* = 1.36, *df* = 5, *P* = 0.23). Thus, the results show that the Bt toxin and RNAi in the pyramids acted independently against SCD.

The results were qualitatively similar between 2015 (field‐grown cotton) and 2016 (greenhouse‐grown cotton) (Figures 3 and 4). Moreover, two‐way ANOVA for the two pyramids against SCD‐r1 showed no significant difference between years in either mortality or development time (Table S2), which implies dominant expression of both traits. Whereas mortality was significantly higher for Bt + JHB than Bt + JHA (*P* = 0.0054), development time did not differ significantly between the two pyramids and the interaction between year and type of pyramid was not significant (Table S2).

### Fitness cost of resistance to Bt cotton

To test for fitness costs associated with resistance to Bt cotton, we compared the performance between SCD‐r1 and SCD on both types of control cotton plants (W0 and GFP). Mortality did not differ between SCD and SCD‐r1 on either of the two types of control cotton, indicating no fitness cost affecting this trait (Figure 3). By contrast, the significantly slower development for SCD‐r1 than SCD on both types of control cotton indicates a fitness cost affecting this trait (Figure 4). The mean percentage increase in development time for SCD‐r1 relative to SCD was 16% on W0 cotton and 15% on GFP cotton (Figure 4).

### Predicted evolution of resistance to Bt cotton, RNAi cotton and Bt + RNAi cotton

**Figure 5:**
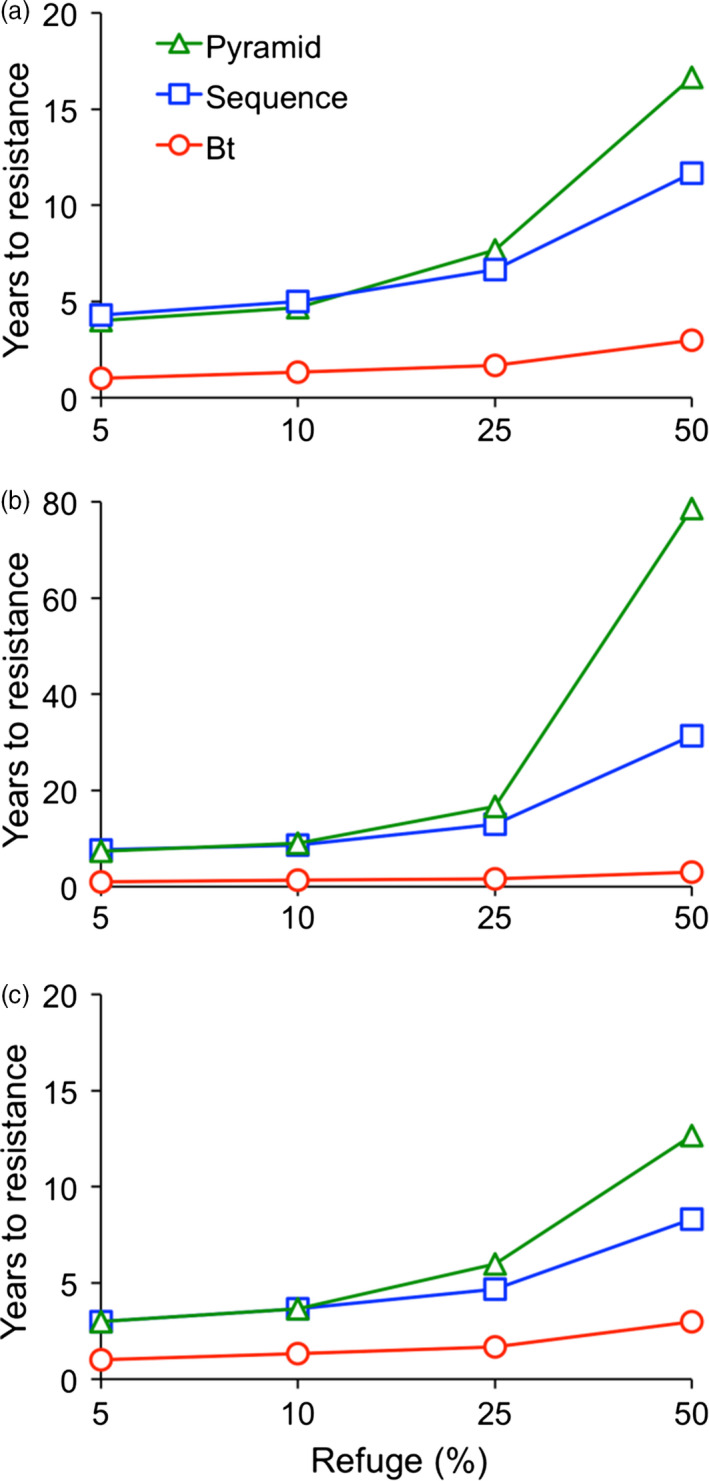
Computer simulations of the evolution of resistance by *H. armigera* to Bt cotton alone, a sequence of Bt cotton followed by RNAi cotton and a pyramid of Bt + RNAi cotton. We used a deterministic model with two alleles (*r* conferring resistance and *s* susceptibility) at each of two independently segregating loci. Locus one controlled survival on Bt cotton and locus two controlled survival on RNAi cotton. The initial resistance allele frequency was 0.05 for Bt cotton and 0.001 for RNAi cotton. The time to resistance is the number of years until the population fitness on transgenic cotton exceeded 0.50. The time to resistance for the sequence is the sum of the time for resistance to Bt cotton and RNAi cotton. (a) Realistic scenario: dominance of resistance (*h*) = 0.5 for Bt cotton and RNAi cotton and no fitness cost. (b) Optimistic scenario: dominance of resistance (*h*) = 0.5 for Bt cotton and 0.2 for RNAi cotton and minor, additive fitness cost. Each *r* allele reduced fitness on non‐transgenic host plants by 0.05; fitness of doubly resistant homozygotes (*r*_1_ *r*_1_ *r*_2_ *r*_2_) on non‐transgenic host plants = 0.80. (c) Pessimistic scenario: identical to (a) except initial resistance allele frequency for resistance to RNAi = 0.01.

We conducted computer simulations of evolution of resistance by *H. armigera* to Bt cotton alone, a sequence of Bt cotton followed by RNAi cotton and a pyramid of Bt + RNAi cotton. We used a deterministic population genetic model where resistance to each type of cotton was controlled by an independently segregating locus with two alleles (Brévault *et al*., 2013). We based key parameters in the model on data available for *H. armigera* in northern China either reported here or previously (Tables S3–S5). We simulated a realistic scenario with no fitness cost and intermediate dominance of resistance for both Bt cotton and RNAi cotton (*h* = 0.5; *h* ranges from 0 for completely recessive resistance to 1 for completely dominant resistance, Liu and Tabashnik, 1997) (Figure 5a), and an optimistic scenario with a minor, additive fitness cost associated with resistance, *h* = 0.5 for Bt cotton, and *h* = 0.2 for RNAi cotton (Figure 5b). We set the initial resistance allele frequency for resistance to Bt cotton at 0.05 based on 2013 bioassay data for *H. armigera* in northern China, which reflects extensive exposure to Bt cotton and a significant increase in resistance over time (Jin *et al*., 2015). We used 0.001 as the value for the initial resistance allele frequency for resistance to RNAi cotton in the realistic and optimistic scenarios, because this is a standard estimate for populations that have not been exposed previously to a particular toxin or control method (Carrière *et al*., 2010). We also evaluated a pessimistic scenario (Figure 5c), which was identical to the realistic scenario (Figure 5a), except the initial resistance allele frequency was 0.01 for resistance to RNAi cotton. We recorded the time to resistance as the number of years until the population fitness on transgenic cotton exceeded 0.50. We focused on a 50% refuge of non‐transgenic host plants, which is slightly less than the previously estimated 56% effective refuge percentage in northern China, including non‐cotton host plants (Jin *et al*., 2015). We also evaluated refuges of 5%, 10% and 25% because smaller refuges are important elsewhere (e.g. in the United States) and might be considered for China in the future.

The simulation results (Figure 5) show that in all cases, increasing the refuge percentage slowed the evolution of resistance, as expected. However, resistance to Bt cotton alone occurred in less than 4 years under all conditions examined. Adding RNAi cotton delayed resistance whether it was used in a sequence (after resistance evolved to Bt cotton) or simultaneously with Bt cotton in a pyramid. The delay caused by RNAi cotton increased as refuge percentage increased, and it was greater when resistance to RNAi cotton was less dominant (compare Figure 5a and b).

With a 50% refuge, relative to Bt cotton alone, adding RNAi cotton in a sequence increased the time to resistance by 9 and 28 years in the realistic and optimistic scenarios, respectively. With a 50% refuge, relative to Bt cotton alone, using RNAi cotton in a pyramid increased the time to resistance by 14 and 75 years in the realistic and optimistic scenarios, respectively. Thus, with a 50% refuge, the pyramid was markedly more durable than the sequence of Bt cotton followed by RNAi cotton. By contrast, with a 5%, 10% or 25% refuge, the pyramid provided little or no advantage relative to the sequence. The years to resistance for the pyramid minus the years to resistance for the sequence with refuges of 5%, 10% and 25% were, respectively, −0.3, −0.3 and 1 in the realistic scenario and −0.3, 0.3 and 4 in the optimistic scenario. The fractional negative values indicate resistance evolved slightly faster to the pyramid than the sequence.

The results under the pessimistic scenario (Figure 5c) and realistic scenario (Figure 5a) are qualitatively similar. However, compared with the realistic scenario, the time to resistance for the sequence and pyramid was shorter by 3 and 4 years, respectively (Figure 5c), because of the higher initial frequency of resistance to RNAi cotton under the pessimistic scenario. With a 50% refuge in the pessimistic scenario, relative to Bt cotton alone, the time to resistance increased by 5 years when RNAi was used in a sequence and by 10 years when RNAi was used in a pyramid (Figure 5c).

## Discussion

As far as we know, this study is the first to report the efficacy of transgenic plant pyramids combining a Bt toxin and RNAi against Bt‐resistant and susceptible strains of a target insect pest. The results show no cross‐resistance to RNAi suppression of enzymes involved in JH synthesis and transport in the SCD‐r1 strain of *H. armigera*, which had greater than 400‐fold resistance to Cry1Ac (Yang *et al*., 2009; Zhang *et al*., 2012) and suffered no increase in mortality or development time on Bt cotton relative to non‐Bt cotton (Figures 3 and 4). Both JHA and JHB cotton were as effective against the resistant strain as its susceptible parent strain SCD (Figures 3 and 4).

Results of cotton leaf bioassays comparing the effects of Bt cotton, RNAi cotton and Bt + RNAi cotton pyramids revealed that Bt cotton and RNAi act independently against the susceptible SCD strain of *H. armigera*. The mortality of susceptible *H. armigera* caused by feeding on dsRNA from *HaJAHMT* was similar in this study with JHA cotton (63%–69%, Figure 3) to previous results (60%) with artificial diet (Asokan *et al*., 2014). Also similar to results from artificial diet experiments (Asokan *et al*., 2014), JHA and JHB cotton interfered with pupation, resulting in malformed pupae that did not yield healthy adults. Because we analysed development time based on the days from hatching to pupation for healthy pupae, the malformed pupae that developed prematurely did not shorten the mean development time for larvae fed on RNAi cotton or Bt + RNAi cotton (Figures 3 and 4).

In related work, artificial diet bioassays with a susceptible strain of the southern corn rootworm, *Diabrotica undecimpunctata howardi*, showed independent action of Bt toxin Cry3Bb and RNAi suppression of a vacuolar‐sorting protein (Levine *et al*., 2015). The observed lack of cross‐resistance and independent action of Bt toxins and RNAi are consistent with their different modes of action (Fishilevich *et al*., 2016). By contrast, cross‐resistance and antagonism are common between the Bt toxins used in pyramids, particularly those with similar amino acid sequences in domains II and III, respectively (Carrière *et al*., 2015, 2016).

In cotton leaf bioassays, the Bt + RNAi pyramids had 90%–91% efficacy against a susceptible strain of *H. armigera* (Figure 3). These data may underestimate the efficacy of the pyramids because larvae were transferred to untreated artificial diet after feeding on cotton leaves for 10 days. In addition, these efficacy values do not reflect the significantly greater development time on pyramids relative to conventional cotton (5 additional days from neonate to pupa; Figure 4), which could increase mortality caused by natural enemies and abiotic factors in the field. To address these and other potential differences between the laboratory and field, it will be important to test the efficacy of the pyramids in the field. Although the available data indicate the specificity and safety of some other applications of RNAi (Levine *et al*., 2015), it will be necessary to test the transgenic cottons described here to determine their effects on non‐target organisms.

The modelling results here imply that pyramids of Bt + RNAi cotton can be much more durable against *H. armigera* than sequences of Bt cotton followed by RNAi cotton if large refuges are present, such as the ca. 50% natural refuges of non‐cotton host plants in northern China (Jin *et al*., 2015) (Figure 5). In simulations with 5%–25% refuges, resistance evolved rapidly to RNAi cotton used either in sequences or in pyramids with Bt cotton (Figure 5). This reflects the bioassay data demonstrating that neither Bt cotton nor RNAi cotton kill at least 97.5% of susceptible larvae (Figure 3), which is required for optimal durability of pyramids (Carrière *et al*., 2015; Roush, 1998). Because resistance to RNAi has not been reported yet in the laboratory or field, the assumptions in the simulations about the genetic basis of resistance to RNAi and associated fitness costs remain to be tested. Thus, the absolute number of years for evolution of resistance to RNAi cotton cannot be predicted rigorously. Nonetheless, the relative rates of evolution of resistance projected for Bt cotton alone, sequences of Bt cotton followed by RNAi cotton and pyramids of Bt + RNAi cotton were similar across the broad range of assumptions incorporated in the three scenarios examined (Figure 5), which suggests these qualitative trends are robust.

We conclude that under some conditions, combining Bt and RNAi in transgenic plant pyramids has great promise for increasing the sustainability of protection against pests. Because of the large refuges of non‐Bt host plants other than cotton in northern China (Jin *et al*., 2015), the pyramids evaluated here could provide substantial benefits to millions of small‐scale farmers in that region. In principle, other pyramids combining RNAi and Bt toxins to which pests are highly susceptible could foster durable pest control, even with smaller refuges.

## Experimental procedures

### Insect strains

We studied three strains of *H. armigera*: a Cry1Ac‐resistant strain (SCD‐r1), its parent susceptible strain (SCD) and an unrelated susceptible strain (96S). 96S was started with adults collected from Henan Province, China in 1996 (Zhang *et al*., 2009). SCD was started with insects from Côte d'Ivoire (Ivory Coast) >30 years ago (Yang *et al*., 2009). Both susceptible strains had been reared in the laboratory on artificial diet without exposure to Bt toxins or insecticides since they were established (Yang *et al*., 2009; Zhang *et al*., 2009). SCD‐r1 was generated by introgression of the *r1* cadherin resistance allele from the Cry1Ac‐resistant strain GYBT into SCD (Yang *et al*., 2009). In diet bioassays, the concentration of Cry1Ac killing 50% of larvae (LC_50_) was >400 times higher for SCD‐r1 than SCD (Yang *et al*., 2009; Zhang *et al*., 2012). We used SCD and SCD‐r1 for all experiments with one exception. We obtained gene sequences from 96S as described in the section below. We maintained larvae at 26 ± 1 °C, 60 ± 10% relative humidity and 14 h light: 10 h dark for rearing and bioassays.

### Amplification, cloning and sequencing of *HaJHAMT* and *HaJHBP*

We extracted total RNA from *H. armigera* using TRIzol (Invitrogen, Carlsbad, CA). First‐strand cDNA was synthesized using the Transcriptor High‐Fidelity cDNA Synthesis Kit (Roche Deutschland Holding GmbH, Mannheim, Germany). From third instars of the susceptible 96S strain of *H. armigera,* we used primer pair P1 to amplify full‐length *HaJHAMT* and primer pairs P2 to P5 to amplify *HaJHBP* (Table S1). We designed degenerate primer pair P2 using sequences from four other moth species (*Bombyx mori*,* Galleria mellonella*,* Manduca sexta* and *Heliothis virescens*, Figure S3). All PCR products were gel‐purified, cloned into the pGEM‐T vector (Promega, Madison, WI, US) and sequenced by Invitrogen (Shanghai, China).

### Transcription of *HaJHAMT* and *HaJHBP* in *H. armigera* larvae

We used quantitative real‐time PCR (qRT‐PCR) to measure transcription of *HaJHAMT* and *HaJHBP* in whole *H. armigera* larvae of different ages (0–11 days after hatching) and in different tissues of fourth instars (head, fat body, midgut and epidermis). First‐strand cDNA was synthesized using Transcriptor High‐Fidelity cDNA Synthesis Kit (Roche), and qRT‐PCR was performed using LightCycler 480 system (Roche) with LightCycler 480 SYBR Green Master, from Roche, following a two‐step protocol: 95 °C for 10 min, 45 cycles of denaturation at 95 °C for 10 s, annealing at 55 °C for 20 s and extension at 72 °C for 20 s. We calculated transcription relative to *actin* with the 2^−ΔCT^ method. Table S1 lists the primers used for qRT‐PCR.

### Transformation of cotton

We constructed vectors that express dsRNA from target gene fragments JHA, JHB or GFP (as a negative control) (Figure 1b) by modifying the plasmid pCAMBIA2300 (CAMBIA, Canberra, Australia) as follows: We inserted the PRP promoter (Xie *et al*., 2001) and the NOS terminator (Depicker *et al*., 1982) into the EcoRI/PstI and PstI/HindIII sites, respectively. The target gene fragments were PCR‐amplified using full‐length larval cDNAs as template. The primers for target gene fragments (Table S1) were designed using GenBank data for *HaJHAMT* (AB127945) and using the sequence obtained here for *HaJHBP* (Figure S3). A 489 bp fragment of the gene encoding GFP was obtained from the plasmid pMD‐GFP (Haseloff *et al*., 1997). Each target gene fragment was inserted into pUCCRNAi, which has two multiclone site regions flanking a 166‐nucleotide intron of the potato GA20‐oxidase gene to facilitate the subcloning of target gene fragments in an inverted repeat manner (Yan *et al*., 2007). Hairpin RNA expression cassettes were also inserted into the PstI site of the modified plasmid to generate each of the three dsRNA‐expressing vectors.

We used each of the three modified plasmids and *Agrobacterium tumefaciens* strain LBA 4404 with plasmid pAL4404 carrying the *vir* and *strep* genes to transform cotton. We generated transgenic plants using *Agrobacterium*‐mediated transformation of the hypocotyledonary axis of axenic cultures of W0 cotton and screened them on solid plates containing 50 mg kanamycin per litre (Figure S8) as described previously (Wu *et al*., 2008a, b).

### PCR and Southern analysis of transgenic lines

For kanamycin‐resistant, putative transgenic cotton lines, we confirmed integration of transgenes using PCR and Southern analysis (Figures S9‐S11). PCR detected the target transgene fragments in the putative transgenic lines, and four of the PCR positive amplicons for each transgene were confirmed by sequencing (Figure S9). For Southern analysis (Figure S10), genomic DNA (15 μg) from PCR positive lines was digested overnight with HindIII enzyme, separated on gels with 0.8% agarose, then transferred onto a Hybond‐N+ membrane (GE Healthcare, Bucks, UK). DNA blot analysis of transgenic cottons was carried out using a probe specific for a portion of the binary vector sequence (Figure S11).

### Transcription of *HaJHAMT* and *HaJHBP*

To measure transcription of *HaJHAMT* and *HaJHBP*, we performed real‐time quantitative PCR (qRT‐PCR) using the LightCycler 480 (Roche) and the HiScript Q RT SuperMix (Vazyme, Nanjing, China), following a two‐step protocol: 95 °C for 10 min, 45 cycles of denaturation at 95 °C for 10 s, annealing at 55 °C for 20 s and extension at 72 °C for 20 s. We used actin as the internal control and calculated transcription relative to *actin* with the 2^−ΔΔCT^ method (Livak and Schmittgen, 2001). The primers used for qRT‐PCR are listed in Table S1.

### Juvenile hormone concentration

We determined the JH concentration in larvae using liquid chromatography–mass spectrometry LC/MS‐8050 (Shimadzu, Kyoto, Japan) as described by Furuta *et al*. (2013), except that we measured JH II rather than JH III. After JHs were extracted with *n*‐hexane, the samples were dried under a stream of nitrogen and redissolved in a 60% (w/v) mixture of acetonitrile and water.

### Cotton leaf bioassays

We tested susceptible (SCD) and Cry1Ac‐resistant (SCD‐r1) larvae of *H. armigera* in bioassays with fully expanded young cotton leaves detached from field‐grown plants in 2015 and from greenhouse‐grown plants in 2016. In 2015, cotton was planted in mid‐April at the Jiangpu Experiment Station of Nanjing Agricultural University in Nanjing, China, and managed with standard agronomic practices including fertilization, irrigation and weed control, but without insecticides. Leaves were removed from cotton plants in late July 2015. In 2016, cotton was planted in the greenhouse at Nanjing Agricultural University (Weigang campus) and grown with 16 h light: 8 h dark, 23 000 lux, 30 ± 5 °C, 60 ± 20% relative humidity, fertilizer once per week and watering twice per week. Leaves were first collected 45 days after planting, when plants had six true leaves.

For bioassays in both years, each cotton leaf was placed in a plastic Petri dish (10 cm diameter by 1.5 cm high) containing a piece of wet filter paper (10 cm diameter) and inoculated with five neonates. The dishes were held in the laboratory at 26 ± 1 °C, 60 ± 10% relative humidity and 14 h light: 10 h dark. After 6 days, we replaced the leaf with a fresh leaf. After 10 days, we transferred larvae to untreated artificial diet and recorded pupation daily from 14 to 30 days after hatching. We calculated survival (%) as the percentage of larvae that became normal pupae (not malformed) by 30 days and mortality (%) as 100% minus survival (%). Malformed pupae were not considered survivors because they do not become viable adults. We calculated the efficacy (%) of transgenic cotton as 100% multiplied by [1 – (survival on transgenic cotton divided by survival on non‐transgenic cotton)], with the untransformed parent cotton (W0) as the non‐transgenic cotton. We calculated development time as the number of days from hatching to pupation for survivors.

In each year, each treatment was replicated three times with 50 larvae per replicate. In both years, we tested seven types of cotton: two controls (W0 and GFP), Bt cotton, two types of RNAi cotton (JHA and JHB) and two pyramids (Bt + JHA and Bt + JHB). Both insect strains were tested on all seven types of cotton in both years, with one exception. The susceptible strain was tested on pyramids in 2016, but not in 2015.

### Computer simulations

To evaluate evolution of resistance by *H. armigera* in northern China to Bt cotton, a sequence of Bt cotton followed by RNAi cotton and a pyramid of Bt + RNAi cotton, we used a previously described, deterministic, two‐locus population genetic model (Brévault *et al*., 2013) with some modifications. We chose this relatively simple model for several reasons: to elucidate how different types of transgenic cotton affect rates of evolution of resistance, to enable incorporation of realistic biological parameters for *H. armigera* in northern China, to examine expected outcomes of different assumptions about dominance and refuges using the same basic model and to make the modelling results readily verifiable by readers. Furthermore, when we used the parameter values of Alstad (2005) and Hamilton (2009) in our model, our model's projections agreed with the projections from their models (Brévault *et al*., 2013).

Our model simulated two unlinked autosomal loci (Brévault *et al*., 2013), similar to the models of Gould (1986), Alstad (2005), Gould *et al*. (2006) and Hamilton (2009). Locus one controlled responses to Bt cotton and locus 2 controlled responses to RNAi cotton. Each locus had two alleles: *r*_1_ and *r*_2_ conferring resistance and *s*_1_ and *s*_2_ susceptibility to Bt cotton and RNAi cotton, respectively. The results reported here show that resistance to Bt cotton does not confer cross‐resistance to RNAi cotton (Figures 3 and 4), thereby supporting the assumption that the loci conferring resistance to Bt cotton and RNAi cotton are different and not linked.

For each year, we simulated three generations, corresponding to the three generations that *H. armigera* feeds on cotton in northern China (Wu and Guo, 2004). As detailed in Data S1 and S2, we based modelling assumptions primarily on empirical data for *H. armigera* in northern China, as in our previous modelling for this pest (Jin *et al*., 2015). Tables S3–S5 summarize the parameter values we examined. We conducted sensitivity analyses to evaluate the impact of variation in assumptions about refuge percentage, fitness cost and dominance (see Data S1 and S2).

#### Time to resistance

After each generation, we calculated the population's fitness on transgenic cotton as the sum of the fitness values of the nine genotypes on transgenic cotton weighted by the proportion of each genotype in the population (Brévault *et al*., 2013). The time to resistance was the number of years until the population's fitness on the transgenic cotton was ≥0.50. We calculated the time to resistance for a sequence of Bt cotton followed by RNAi cotton as the time for resistance to Bt cotton plus the time for resistance to RNAi cotton.

### Statistical analyses

We used one‐way analysis of variance (ANOVA) with Tukey's honestly significant difference (HSD) to test for significant differences (*P* < 0.05) between treatments in relative transcription (Figure 2a,b), JH concentration (Figure 2c), as well as mortality and development time within strains of *H. armigera* (Figures 3 and 4) (http://statistica.mooo.com/OneWay_Anova_with_TukeyHSD). We used *t*‐tests to assess the *a priori* null hypothesis that no difference occurred between the resistant (SCD‐r1) and susceptible (SCD) strains of *H. armigera* in mortality or development time on each of the seven types of cotton leaves tested in bioassays (Figures 3 and 4). To test for independent action of the Bt toxin and RNAi on survival of SCD in cotton leaf bioassays, we used a one‐sample *t*‐test to determine whether the observed values for the IMS differed significantly from 1, and *t*‐test was performed using VassarStats software (http://vassarstats.net/). We used two‐way ANOVA to test for effects of year (2015 vs. 2016), type of pyramid (JHA + Bt vs. JHB + Bt) and their interaction on mortality and development time for SCD‐r1 also using VassarStats.

## Conflict of interest

Z.Z. and Xiaofang Wang have a Chinese patent application related to this work, number CN102464710A, ‘*Helicoverpa armigera* juvenile hormone‐binding protein (HaJHBP) and encoding gene and application thereof’. B.E.T. is co‐author of a patent on modified Bt toxins, ‘Suppression of Resistance in Insects to *Bacillus thuringiensis* Cry Toxins, Using Toxins that do not Require the Cadherin Receptor’ (patent numbers: CA2690188A1, CN101730712A, EP2184293A2,EP2184293A4, EP2184293B1, WO2008150150A2, WO2008150150A3). DuPont Pioneer, Dow AgroSciences, Monsanto, Bayer CropScience and Syngenta did not provide funding to support this work, but may be affected financially by publication of this paper and have funded other work by B.E.T.

## Supporting information


**Figure S1.** cDNA sequence alignment of *JHAMT* for seven insect species.
**Figure S2.** Amino acid sequence alignment of JHAMT for eight insect species.
**Figure S3.** cDNA sequence alignment of *JHBP* for six insect species.
**Figure S4.** Amino acid sequence alignment of JHBP for ten insect species.
**Figure S5.** Transcription of *HaJHAMT* and *HaJHBP* in whole *H. armigera* larvae of different ages.
**Figure S6.** Transcription of *HaJHAMT* and *HaJHBP* in different tissues of fourth instars of *H. armigera*.
**Figure S7.** Efficacy of JHA and JHB dsRNA in artificial diet against *H. armigera* larvae.
**Figure S8.** Development of transgenic cotton by *Agrobacterium*‐mediated transformation.
**Figure S9.** PCR detection of transgenic T_3_ cotton plants.
**Figure S10.** Southern blot detection of transgenic T_3_ plants.
**Figure S11.** Probe sequence for Southern analysis of transgenic cotton that matches a portion of the binary vector sequence.
**Table S1.** Primers used in this study.
**Table S2.** Two‐way ANOVA: effects of year (2015 vs. 2016) and type of pyramid (Bt + JHA vs. Bt + JHB) on mortality and development time of resistant strain SCD‐r1 of *H. armigera* in cotton leaf bioassays.
**Table S3.** Parameter values used in simulations.
**Table S4.** Fitness of the nine *H. armigera* genotypes on pyramided Bt + RNAi cotton in simulations as a function of dominance of resistance to the pyramid (*hp*).
**Table S5.** Fitness of the nine *H. armigera* genotypes on refuge plants in simulations of pyramided Bt + RNAi cotton with a minor, additive fitness cost.
**Data S1.** Methods.
**Data S2.** References.

## References

[pbi12709-bib-0001] Alstad, D. (2005) Populus Java Version 5.4. Department of Ecology, Evolution, and Behavior, University of Minnesota. Available at: http://www.cbs.umn.edu/populus [Accessed 2 September, 2016].

[pbi12709-bib-0002] Asokan, R. , Sharath Chandra, G. , Manamohan, M. , Krishna Kumar, N.K. and Sita, T. (2014) Response of various target genes to diet‐delivered dsRNA mediated RNA interference in the cotton bollworm, *Helicoverpa armigera* . J. Pest Sci. 87, 163–172.

[pbi12709-bib-0003] Baum, J.A. and Roberts, J.K. (2014) Progress towards RNAi‐mediated pest management. Adv. Insect Physiol. 47, 249–295.

[pbi12709-bib-0004] Baum, J.A. , Bogaert, T. , Clinton, W. , Heck, G.R. , Feldmann, P. , Ilagan, O. , Johnson, S. *et al*. (2007) Control of coleopteran insect pests through RNA interference. Nat. Biotechnol. 25, 1322–1326. 17982443 10.1038/nbt1359

[pbi12709-bib-0006] Belles, X. , Martin, D. and Piulachs, M.D. (2005) The mevalonate pathway and the synthesis of juvenile hormone in insects. Annu. Rev. Entomol. 50, 181–199. 15355237 10.1146/annurev.ento.50.071803.130356

[pbi12709-bib-0007] Brévault, T. , Heuberger, S. , Zhang, M. , Ellers‐Kirk, C. , Ni, X. , Masson, L. , Li, X. *et al*. (2013) Potential shortfall of pyramided transgenic cotton for insect resistance management. Proc. Natl Acad. Sci. USA, 110, 5806–5811. 23530245 10.1073/pnas.1216719110 PMC3625267

[pbi12709-bib-0009] Carrière, Y. , Crowder, D.W. and Tabashnik, B.E. (2010) Evolutionary ecology of insect adaptation to Bt crops. Evol. Appl. 3, 561–573. 25567947 10.1111/j.1752-4571.2010.00129.x PMC3352503

[pbi12709-bib-0010] Carrière, Y. , Crickmore, N. and Tabashnik, B.E. (2015) Optimizing pyramided transgenic Bt crops for sustainable pest management. Nat. Biotechnol. 33, 161–168. 25599179 10.1038/nbt.3099

[pbi12709-bib-0011] Carrière, Y. , Fabrick, J.A. and Tabashnik, B.E. (2016) Can pyramids and seed mixtures delay resistance to Bt crops? Trends Biotechnol. 34, 291–302. 26774592 10.1016/j.tibtech.2015.12.011

[pbi12709-bib-0012] Depicker, A. , Stachel, S. , Dhaese, P. , Zambryski, P. and Goodman, H.M. (1982) Nopaline synthase: transcript mapping and DNA sequence. J. Mol. Appl. Genet. 1, 561–573. 7153689

[pbi12709-bib-0013] Farias, J.R. , Andow, D.A. , Horikoshi, R.J. , Sorgatto, R.J. , Fresia, P. , dos Santos, A.C. and Omoto, C. (2014) Field‐evolved resistance to Cry1F maize by *Spodoptera frugiperda* in Brazil. Crop Prot. 64, 150–158.

[pbi12709-bib-0014] Fishilevich, E. , Vélez, A.M. , Storer, N.P. , Li, H. , Bowling, A.J. , Rangasamy, M. , Worden, S.E. *et al*. (2016) RNAi as a management tool for the western corn rootworm, *Diabrotica virgifera virgifera* . Pest Manag. Sci., 72, 1652–1663. 27218412 10.1002/ps.4324

[pbi12709-bib-0015] Fu, K.Y. , Li, Q. , Zhou, L.T. , Meng, Q.W. , Lü, F.G. , Guo, W.C. and Li, G.Q. (2016) Knockdown of *juvenile hormone acid methyl transferase* severely affects the performance of *Leptinotarsa decemlineata* (Say) larvae and adults. Pest Manag. Sci. 72, 1231–1241. 26299648 10.1002/ps.4103

[pbi12709-bib-0016] Furuta, K. , Ichikawa, A. , Murata, M. , Kuwano, E. , Shinoda, T. and Shiotsuki, T. (2013) Determination by LC‐MS of juvenile hormone titers in hemolymph of the silkworm, *Bombyx mori* . Biosci. Biotechnol. Biochem. 77, 988–991. 23649254 10.1271/bbb.120883

[pbi12709-bib-0017] Gassmann, A.J. , Petzold‐Maxwell, J.L. , Clifton, E.H. , Dunbar, M.W. , Hoffmann, A.M. , Ingber, D.A. and Keweshan, R.S. (2014) Field‐evolved resistance by western corn rootworm to multiple *Bacillus thuringiensis* toxins in transgenic maize. Proc. Natl Acad. Sci. USA, 111, 5141–5146. 24639498 10.1073/pnas.1317179111 PMC3986160

[pbi12709-bib-0018] Gould, F. (1986) Simulation models for predicting durability of insect‐resistant germ plasm: a deterministic diploid, two‐locus model. Environ. Entomol. 15, 1–10.

[pbi12709-bib-0019] Gould, F. , Cohen, M.B. , Bentur, J.S. , Kennedy, G.G. and Van Duyn, J. (2006) Impact of small fitness costs on pest adaptation to crop varieties with multiple toxins: a heuristic model. J. Econ. Entomol. 99, 2091–2099. 17195678 10.1603/0022-0493-99.6.2091

[pbi12709-bib-0020] Guo, S. , Cui, H. , Xu, Q. and Ni, W. (1995). Expressive carrier with coded insect‐killing protein fusion gene, and transfer gene plant. Chinese Patent Pub. No. CN1134981.

[pbi12709-bib-0021] Hamilton, M.B. (2009) Population Genetics. Wiley‐Blackwell, Hoboken, NJ. 407 pp. Interact Box 2.5 Spreadsheet Simulation. Available at: http://www.blackwellpublishing.com/ hamiltongenetics/chapter_resources.asp [Accessed September 2, 2016]

[pbi12709-bib-0022] Haseloff, J. , Siemering, K.R. , Prasher, D.C. and Hodge, S. (1997) Removal of a cryptic intron and subcellular localization of green fluorescent protein are required to mark transgenic *Arabidopsis* plants brightly. Proc. Natl Acad. Sci. USA, 94, 2122–2127. 9122158 10.1073/pnas.94.6.2122 PMC20051

[pbi12709-bib-0023] Hutchison, W.D. , Burkness, E.C. , Mitchell, P.D. , Moon, R.D. , Leslie, T.W. , Fleischer, S.J. , Abrahamson, M. *et al*. (2010) Areawide suppression of European corn borer with Bt maize reaps savings to non‐Bt maize growers. Science, 330, 222–225. 20929774 10.1126/science.1190242

[pbi12709-bib-0024] Jakka, S.R.K. , Shreshtha, R.B. and Gassmann, A.J. (2016) Broad‐spectrum resistance to *Bacillus thuringiensis* toxins by western corn rootworm *(Diabrotica virgifera virgifera)* . Sci. Rep. 6, 27860. 27297953 10.1038/srep27860 PMC4906537

[pbi12709-bib-0025] James, C. (2016) Twentieth Anniversary (1996 to 2015) of the Global Commercialization of Biotech Crops and Biotech Crop Highlights in 2015. ISAAA Briefs No. 51. Ithaca, NY: ISAAA.

[pbi12709-bib-0026] Jin, L. , Zhang, H. , Lu, Y. , Yang, Y. , Wu, K. , Tabashnik, B.E. , Wu, Y. (2015) Large‐scale test of the natural refuge strategy for delaying insect resistance to transgenic Bt crops. Nat. Biotechnol. 33, 169–174. 25503384 10.1038/nbt.3100

[pbi12709-bib-0027] Kagan, R.M. and Clarke, S. (1994) Widespread occurrence of three sequence motifs in diverse S‐adenosylmethionine‐dependent methyltransferases suggests a common structure for these enzymes. Arch. Biochem. Biophys. 310, 417–427. 8179327 10.1006/abbi.1994.1187

[pbi12709-bib-0028] Kim, Y.H. , Soumaila Issa, M. , Cooper, A.M.W. and Zhu, K.Y. (2015) RNA interference: applications and advances in insect toxicology and insect pest management. Pestic. Biochem. Physiol. 120, 109–117. 25987228 10.1016/j.pestbp.2015.01.002

[pbi12709-bib-0029] Levine, S.L. , Tan, J. , Mueller, G.M. , Bachman, P.M. , Jensen, P.D. and Uffman, J.P. (2015) Independent action between DvSnf7 RNA and Cry3Bb1 protein in southern corn rootworm, *Diabrotica undecimpunctata howardi* and Colorado potato beetle. *Leptinotarsa decemlineata* . PLoS ONE, 10, e0118622. 25734482 10.1371/journal.pone.0118622 PMC4348175

[pbi12709-bib-0030] Lim, Z.X. , Robinson, K.E. , Jain, R.G. , Chandra, G.S. , Asokan, R. , Asgari, S. and Mitter, N. (2016) Diet‐delivered RNAi in *Helicoverpa armigera* – Progresses and challenges. J. Insect Physiol. 85, 86–93. 26549127 10.1016/j.jinsphys.2015.11.005

[pbi12709-bib-0031] Liu, Y. and Tabashnik, B.E. (1997) Inheritance of resistance to the *Bacillus thuringiensis* toxin Cry1C in the diamondback moth. Appl. Environ. Microbiol. 63, 2218–2223. 16535623 10.1128/aem.63.6.2218-2223.1997 PMC1389178

[pbi12709-bib-0032] Livak, K.J. and Schmittgen, T.D. (2001) Analysis of relative gene expression data using real‐time quantitative PCR and the 2^−ΔΔCT^ method. Methods, 25, 402–408. 11846609 10.1006/meth.2001.1262

[pbi12709-bib-0033] Lu, Y. , Wu, K. , Jiang, Y. , Guo, Y. and Desneux, N. (2012) Widespread adoption of Bt cotton and insecticide decrease promotes biocontrol services. Nature, 487, 362–365. 22722864 10.1038/nature11153

[pbi12709-bib-0034] Mao, Y.B. , Cai, W.J. , Wang, J.W. , Hong, G.J. , Tao, X.Y. , Wang, L.J. , Huang, Y.P. *et al*. (2007) Silencing a cotton bollworm P450 monooxygenase gene by plant‐mediated RNAi impairs larval tolerance of gossypol. Nat. Biotechnol. 25, 1307–1313. 17982444 10.1038/nbt1352

[pbi12709-bib-0035] Minakuchi, C. , Namiki, T. , Yoshiyama, M. and Shinoda, T. (2008) RNAi‐mediated knockdown of juvenile hormone acid O‐methyltransferase gene causes precocious metamorphosis in the red flour beetle *Tribolium castaneum* . FEBS J. 275, 2919–2931. 18435763 10.1111/j.1742-4658.2008.06428.x

[pbi12709-bib-0036] National Academies of Sciences, Engineering, and Medicine (NASEM) . (2016) Genetically Engineered Crops: Experiences and Prospects. Washington: National Academies Press. 28230933

[pbi12709-bib-0037] Pietrzyk, A.J. , Jaskolski, M. and Bujacz, G. (2013) Structural studies of juvenile hormone binding proteins. In Juvenile Hormones and Juvenoids: Modeling Biological Effects and Environmental Fate ( Devillers, J. , ed), pp. 291–309. Boca Raton: CRC Press.

[pbi12709-bib-0038] Roush, R.T. (1998) Two‐toxin strategies for management of insecticidal transgenic crops: can pyramiding succeed where pesticide mixtures have not?. Phil. Trans. Roy. Soc. B Biol. Sci. 353, 1777–1786.

[pbi12709-bib-0039] Shinoda, T. and Itoyama, K. (2003) Juvenile hormone acid methyltransferase: a key regulatory enzyme for insect metamorphosis. Proc. Natl Acad. Sci. USA, 100, 11986–11991. 14530389 10.1073/pnas.2134232100 PMC218700

[pbi12709-bib-0040] Suzuki, R. , Fujimoto, Z. , Shiotsuki, T. , Tsuchiya, W. , Momma, M. , Tase, A. , Miyazawa, M. *et al*. (2011) Structural mechanism of JH delivery in hemolymph by JHBP of silkworm, Bombyx mori. Sci. Rep. 1, 133. 22355650 10.1038/srep00133 PMC3216614

[pbi12709-bib-0041] Tabashnik, B.E. (2016) Tips for battling billion‐dollar beetles. Science, 354, 552–553. 27811255 10.1126/science.aag101

[pbi12709-bib-0042] Tabashnik, B.E. , Sisterson, M.S. , Ellsworth, P.C. , Dennehy, T.J. , Antilla, L. , Liesner, L. , Whitlow, M. *et al*. (2010) Suppressing resistance to Bt cotton with sterile insect releases. Nat. Biotechnol. 28, 1304–1307. 21057498 10.1038/nbt.1704

[pbi12709-bib-0043] Tabashnik, B.E. , Wu, K. and Wu, Y. (2012) Early detection of field‐evolved resistance to Bt cotton in China: cotton bollworm and pink bollworm. J. Invert. Pathol. 110, 301–306. 10.1016/j.jip.2012.04.008 22537835

[pbi12709-bib-0044] Tabashnik, B.E. , Brévault, T. and Carrière, Y. (2013) Insect resistance to Bt crops: lessons from the first billion acres. Nat. Biotechnol. 31, 510–521. 23752438 10.1038/nbt.2597

[pbi12709-bib-0045] Tay, W.T. , Soria, M.F. , Walsh, T. , Thomazoni, D. , Silvie, P. , Behere, G.T. , Anderson, C. *et al*. (2013) A brave new world for an Old World pest: *Helicoverpa armigera* (Lepidoptera: Noctuidae) in Brazil. PLoS ONE, 8, e80134. 24260345 10.1371/journal.pone.0080134 PMC3832445

[pbi12709-bib-0046] Tian, G. , Cheng, L. , Qi, X. , Ge, Z. , Niu, C. , Zhang, X. and Jin, S. (2015) Transgenic cotton plants expressing double‐stranded RNAs target HMG‐CoA reductase (*HMGR*) gene inhibits the growth, development and survival of cotton bollworms. Intl. J. Biol. Sci. 11, 1296–1305. 10.7150/ijbs.12463 PMC4582153 26435695

[pbi12709-bib-0048] Wan, P. , Zhang, Y. , Wu, K. and Huang, M. (2005) Seasonal expression profiles of insecticidal protein and control efficacy against *Helicoverpa armigera* for Bt cotton in the Yangtze River Valley of China. J. Econ. Entomol. 98, 195–201. 15765683 10.1093/jee/98.1.195

[pbi12709-bib-0049] Wojtasek, H. and Prestwich, G.D. (1995) Key disulfide bonds in an insect hormone binding protein: cDNA cloning of a juvenile hormone binding protein of *Heliothis virescens* and ligand binding by native and mutant forms. Biochemistry, 34, 5234–5241. 7711043 10.1021/bi00015a037

[pbi12709-bib-0050] Wu, Y. (2014) Detection and mechanisms of resistance evolved in insects to Cry toxins from *Bacillus thuringiensis* . Adv. Insect Physiol. 47, 297–342.

[pbi12709-bib-0051] Wu, K.M. and Guo, Y.Y. (2004) The evolution of cotton pest management practices in China. Annu. Rev. Entomol. 50, 31–52. 10.1146/annurev.ento.50.071803.130349 15355239

[pbi12709-bib-0052] Wu, K.M. , Lu, Y.H. , Feng, H.Q. , Jiang, Y.Y. and Zhao, J.Z. (2008a) Suppression of cotton bollworm in multiple crops in China in areas with Bt toxin‐containing cotton. Science, 321, 1676–1678. 18801998 10.1126/science.1160550

[pbi12709-bib-0053] Wu, S.J. , Wang, H.H. , Li, F.F. , Chen, T.Z. , Zhang, J. , Jiang, Y.J. , Ding, Y.Z. *et al*. (2008b) Enhanced *Agrobacterium*‐mediated transformation of embryogenic calli of upland cotton via efficient selection and timely subculture of somatic embryos. Plant Mol. Biol. Rep. 26, 174–185.

[pbi12709-bib-0054] Xie, Y. , Liu, Y. and Zhu, Z. (2001) Expressing activity of promoter elements of large intergenic region from cotton leaf curl virus in host plant. Sci. China C Life Sci. 44, 8–17. 18763083 10.1007/BF02882067

[pbi12709-bib-0055] Yan, P.Q. , Bai, X.Q. , Wan, X.Q. , Guo, Z.K. , Li, L.J. , Gong, H.Y. and Chu, C.C. (2007) Expression of TMV coat protein gene RNAi in transgenic tobacco plants confer immunity to tobacco mosaic virus infection. Hereditas, 29, 1018–1022. 17681934 10.1360/yc-007-1018

[pbi12709-bib-0056] Yang, Y.H. , Yang, Y.J. , Gao, W.Y. , Guo, J.J. , Wu, Y.H. and Wu, Y.D. (2009) Introgression of a disrupted cadherin gene enables susceptible *Helicoverpa armigera* to obtain resistance to *Bacillus thuringiensis* toxin Cry1Ac. Bull. Entomol. Res. 99, 175–181. 18954492 10.1017/S0007485308006226

[pbi12709-bib-0057] Yu, X.D. , Liu, Z.C. , Huang, S.L. , Chen, Z.Q. , Sun, Y.W. , Duan, P.F. , Ma, Y.Z. *et al*. (2016) RNAi‐mediated plant protection against aphids. Pest Manag. Sci. 72, 1090–1098. 26888776 10.1002/ps.4258

[pbi12709-bib-0058] Zhang, Z.L. , Ni, W.C. , Guo, S.D. , Shu, C.E. , Zhang, B.L. and Xu, Y.J. (1998) Transfer of a synthetic Bt gene to cotton plants. Jiangsu J. Agr. Sci. 14, 145–148.

[pbi12709-bib-0059] Zhang, S. , Cheng, H. , Gao, Y. , Wang, G. , Liang, G. and Wu, K. (2009) Mutation of an aminopeptidase N gene is associated with *Helicoverpa armigera* resistance to *Bacillus thuringiensis* Cry1Ac toxin. Insect Biochem. Mol. Biol. 39, 421–429. 19376227 10.1016/j.ibmb.2009.04.003

[pbi12709-bib-0060] Zhang, H. , Tian, W. , Zhao, J. , Jin, L. , Yang, J. , Liu, C. , Yang, Y. *et al*. (2012) Diverse genetic basis of field‐evolved resistance to Bt cotton in cotton bollworm from China. Proc. Natl Acad. Sci. USA, 109, 10275–10280. 22689968 10.1073/pnas.1200156109 PMC3387040

[pbi12709-bib-0061] Zukoff, S.N. , Ostlie, K.R. , Potter, B. , Meihls, L.N. , Zukoff, A.L. , French, L. , Ellersieck, M.R. *et al*. (2016) Multiple assays indicate varying levels of cross resistance in Cry3Bb1‐selected field populations of the western corn rootworm to mCry3A, eCry3.1Ab, and Cry34/35Ab1. J. Econ. Entomol. 109, 1387–1398. 27106225 10.1093/jee/tow073

