# Supplementary material for: Next‐generation transgenic cotton: pyramiding RNAi and Bt counters insect resistance
Source: Plant Biotechnol J. 2017 Mar 16;15(9):1204–13. doi: 10.1111/pbi.12709 (PMC5552478; doi:10.1111/pbi.12709)
Supplement: Supplementary file 1 — Figure S1. cDNA sequence alignment of JHAMT for seven insect species. Figure S2. Amino acid sequence alignment of JHAMT for eight insect species. Figure S3. cDNA sequence alignment of JHBP for six insect species. Figure S4. Amino acid sequence alignment of JHBP for ten insect species. Figure S5. Transcription of HaJHAMT and HaJHBP in whole H. armigera larvae of different ages. Figure S6. Transcription of HaJHAMT and HaJHBP in different tissues of fourth instars of H. armigera. Figure S7. Efficacy of JHA and JHB dsRNA in artificial diet against H. armigera larvae. Figure S8. Development of transgenic cotton by Agrobacterium‐mediated transformation. Figure S9. PCR detection of transgenic T3 cotton plants. Figure S10. Southern blot detection of transgenic T3 plants. Figure S11. Probe sequence for Southern analysis of transgenic cotton that matches a portion of the binary vector sequence. Table S1. Primers used in this study. Table S2. Two‐way ANOVA: effects of year (2015 vs. 2016) and type of pyramid (Bt + JHA vs. Bt + JHB) on mortality and development time of resistant strain SCD‐r1 of H. armigera in cotton leaf bioassays. Table S3. Parameter values used in simulations. Table S4. Fitness of the nine H. armigera genotypes on pyramided Bt + RNAi cotton in simulations as a function of dominance of resistance to the pyramid (hp). Table S5. Fitness of the nine H. armigera genotypes on refuge plants in simulations of pyramided Bt + RNAi cotton with a minor, additive fitness cost. Data S1. Methods. Data S2. References. [file PBI-15-1204-s001.pdf]

Supporting information

Next-generation transgenic cotton: pyramiding RNAi and Bt counters insect resistance

Mi Ni[1,a], Wei Ma[2,a], Xiaofang Wang[1,a], Meijing Gao[3,a], Yan Dai[1], Xiaoli Wei[1], Lei Zhang[1], Yonggang Peng[1], Shuyuan Chen[1], Lingyun Ding[2], Yue Tian[2], Jie Li[2], Haiping Wang[2], Xiaolin Wang[4], Guowang Xu[4], Wangzhen Guo[2], Yihua Yang[3], Yidong Wu[3], Shannon Heuberger[5], Bruce E. Tabashnik[5*], Tianzhen Zhang[2*] and Zhen Zhu[1*]

[1]State Key Laboratory of Plant Genomics and National Center for Plant Gene Research (Beijing), Institute of Genetics and Developmental Biology, Chinese Academy of Sciences, Beijing, China

[2]National Key Laboratory for Crop Genetics and Germplasm Enhancement, Jiangsu Plant Gene Engineering Research Center, Nanjing Agricultural University, Nanjing, China

[3]College of Plant Protection, Nanjing Agricultural University, Nanjing, China

[4]Key Laboratory of Separation Science for Analytical Chemistry, Dalian Institute of Chemical Physics, Chinese Academy of Sciences, Dalian, China

[5]Department of Entomology, University of Arizona, Tucson, AZ, USA

[a]These authors contributed equally to this work.

*Correspondence should be addressed to Z.Z. (Tel/fax +86 10 64806616; email zzhu@genetics.ac.cn), T.Z. (cotton@njau.edu.cn) and B.E.T (brucet@cals.arizona.edu).

Supporting information

Figure S1. cDNA sequence alignment of *JHAMT* for seven insect species.

Figure S2. Amino acid sequence alignment of JHAMT for eight insect species.

Figure S3. cDNA sequence alignment of *JHBP* for six insect species.

Figure S4. Amino acid sequence alignment of JHBP for ten insect species.

Figure S5. Transcription of *HaJHAMT* and *HaJHBP* in whole *H. armigera* larvae of different ages.

Figure S6. Transcription of *HaJHAMT* and *HaJHBP* in different tissues of fourth instars of *H. armigera*.

Figure S7. Efficacy of JHA and JHB dsRNA in artificial diet against *H. armigera* larvae.

Figure S8. Development of transgenic cotton by *Agrobacterium*-mediated transformation.

Figure S9. PCR detection of transgenic T_3 cotton plants.

Figure S10. Southern blot detection of transgenic T_3 plants.

Figure S11. Probe sequence for Southern analysis of transgenic cotton that matches a portion of the binary vector sequence.

Table S1. Primers used in this study.

Table S2. Two-way ANOVA: Effects of year (2015 vs. 2016) and type of pyramid (Bt + JHA vs. Bt + JHB) on mortality and development time of resistant strain SCD-r1 of *H. armigera* in cotton leaf bioassays.

Table S3. Parameter values used in simulations.

Table S4. Fitness of the nine *H. armigera* genotypes on pyramided Bt + RNAi cotton in simulations as a function of dominance of resistance to the pyramid (*hp*).

Table S5. Fitness of the nine *H. armigera* genotypes on refuge plants in simulations of pyramided Bt + RNAi cotton with a minor, additive fitness cost.

Data S1. Methods.

Data S2. References.

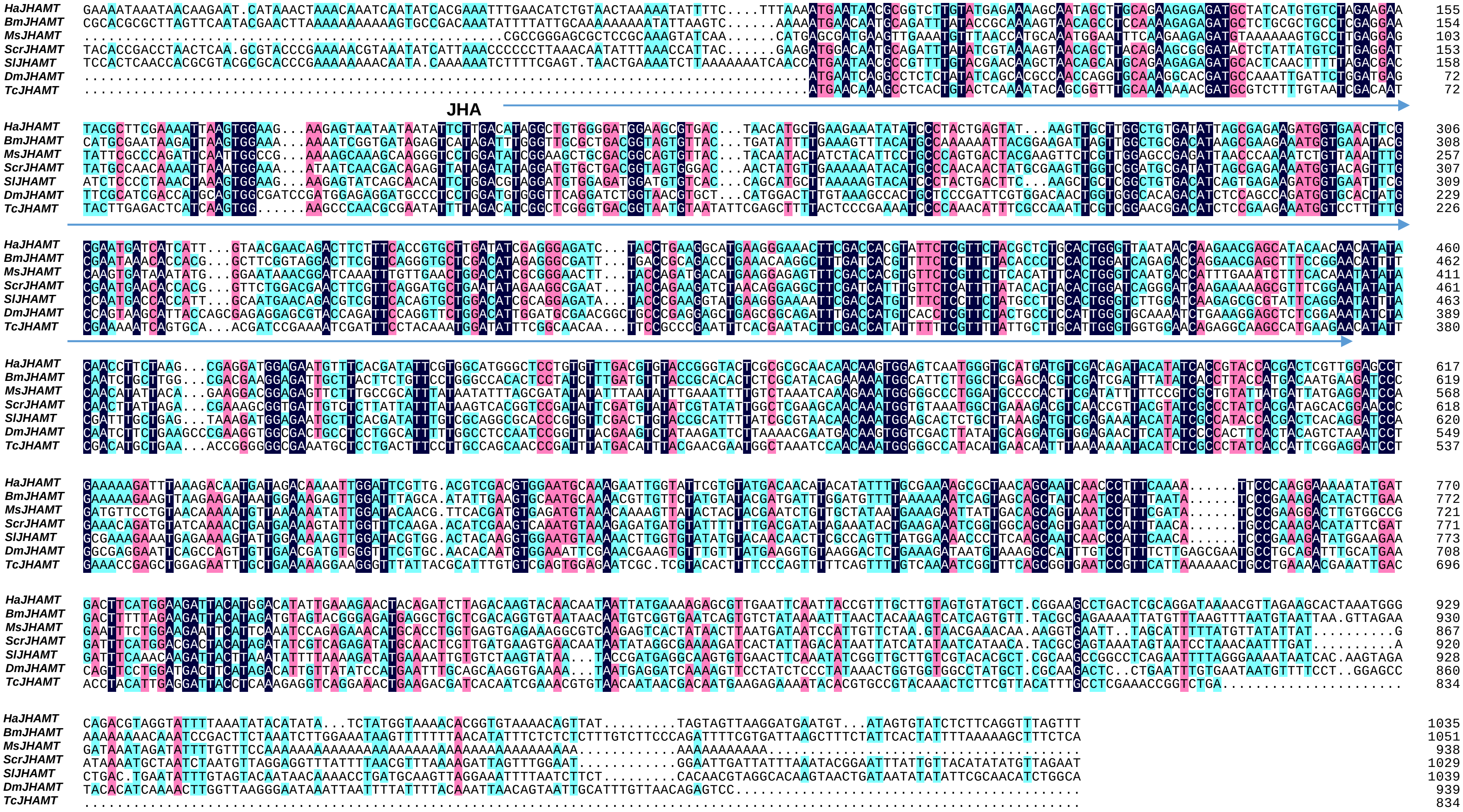

Figure S1. cDNA sequence alignment of *JHAMT* for seven insect species. Five species are Lepidoptera: *HaJHAMT*, *Helicoverpa armigera* (GenBank KX289532.1); *BmJHAMT*, *Bombyx mori* (AB113578.1); *MsJHAMT, Manduca sexta* (AF117590.1); *ScrJHAMT*, *Samia cynthia ricini* (DQ465408.1); *SlJHAMT, Spodoptera litura* (AB127944.1). *DmJHAMT*, *Drosophila melanogaster* (AB113579.1, Diptera) and *TcJHAMT*, *Tribolium castaneum* (NM_001127311.1, Coleoptera). *HaJHAMT* is most closely related to *SlJHAMT* with 73.0% homology. Conservation across species is shown by shading; black for base pairs conserved across all seven species (100%), pink for six species (86%), blue for four or five species (57 or 71%). The blue lines indicate the fragment JHA. Dots indicate artificial gaps introduced to maximize nucleotide matching. Alignments were performed using DNAMAN (ver. 8.0, Lynnon Biosoft).

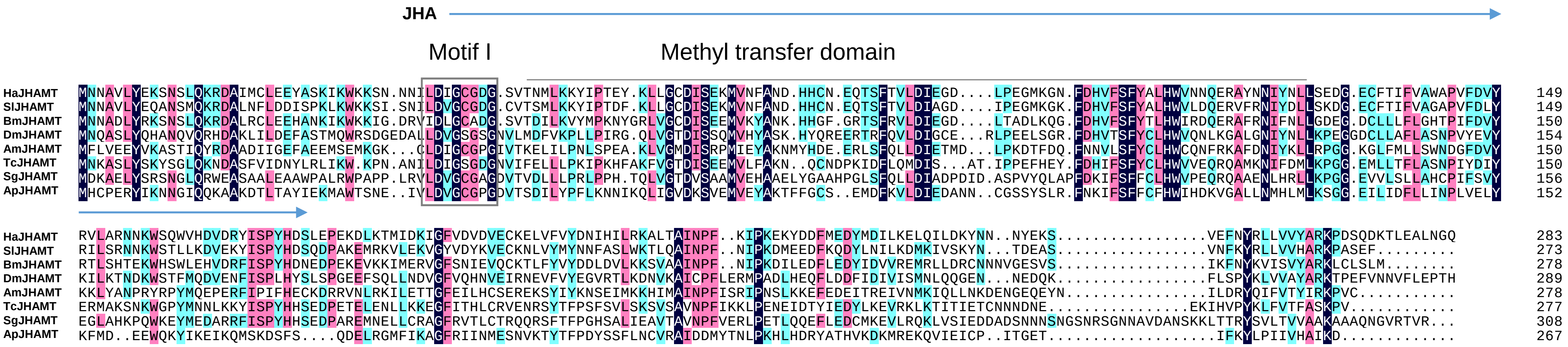

Figure S2. Amino acid sequence alignment of JHAMT for eight insect species. Three species are Lepidoptera: HaJHAMT, *Helicoverpa armigera* (GenBank ANI21405); *SlJHAMT, Spodoptera litura* (BAF63629.1); *BmJHAMT*, *Bombyx mori* (BAC98835.1); DmJHAMT, *Drosophila melanogaster* (NP_609793.2, Diptera); AmJHAMT, *Apis mellifera* (NP_001314896.1, Hymenoptera); TcJHAMT, *Tribolium castaneum* (NP_001120783.1, Coleoptera); SgJHAMT, *Schistocerca gregaria* (ADV17350.1, Orthoptera) and ApJHAMT, *Acyrthosiphon pisum* (NP_001156251.1, Homoptera). The gray box indicates Motif I for SAM binding (Kagan and Clarke, 1994), which is conserved across species. The gray line indicates the conserved methyl transferase domain. Conservation across species is shown by shading; black for amino acids conserved across all seven species (100%), pink for six or seven species (75 or 88%), blue for four or five species (50 or 62%). The blue line indicates the fragment JHA. Dots indicate artificial gaps introduced to maximize nucleotide matching. Alignments were performed using DNAMAN (ver. 8.0, Lynnon Biosoft).

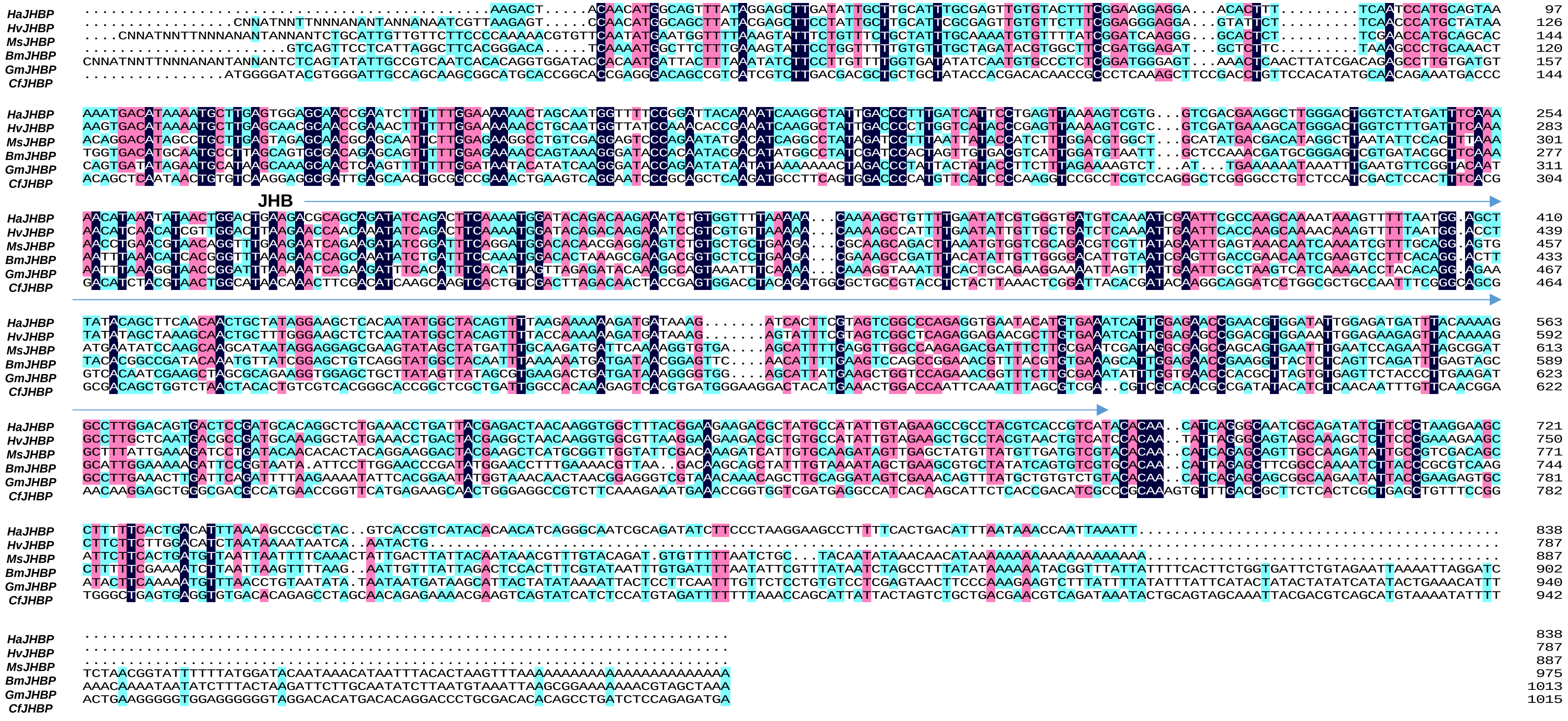

Figure S3. cDNA sequence alignment of *JHBP* for six insect species. Five species are Lepidoptera: *HaJHBP*, *Helicoverpa armigera* (GenBank KX289533.1); *HvJHBP*, *Heliothis virescens* (U22515.1); *MsJHBP*, *Manduca sexta* (S56567.1); *BmJHBP*, *Bombyx mori* (NM_001043609.2); GmJHBP, *Galleria mellonella* (AF410772.3). CfJHBP, *Coptotermes formosanus* (KC571945.1, Blattodea). *HaJHBP* is most closely related to *HvJHBP* with 74.5% homology. Conservation across species is shown by shading; black for base pairs conserved across all six species (100%), pink for five species (83%), blue for three or four species (50 or 67%). The blue lines indicate the fragment JHB. Dots indicate artificial gaps introduced to maximize nucleotide matching. Alignments were performed using DNAMAN (ver. 8.0, Lynnon Biosoft).

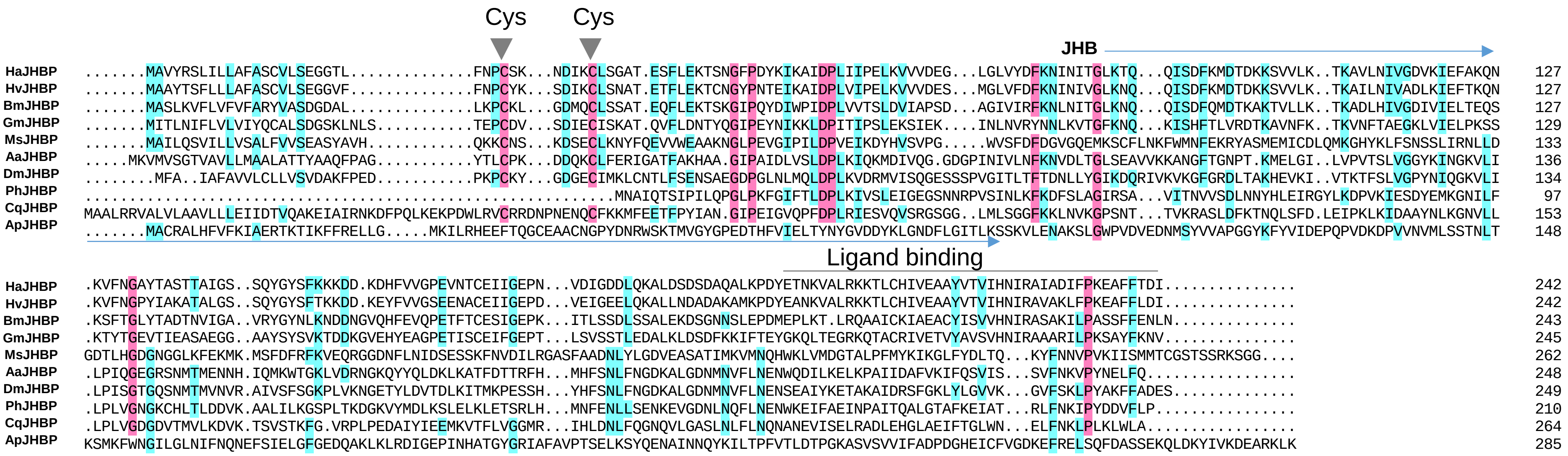

Figure S4. Amino acid sequence alignment of JHBP for ten insect species. Five species are Lepidoptera: HaJHBP, *Helicoverpa armigera* (GenBank ANI21406.1); HvJHBP, *Heliothis virescens* (AAA68242.1); BmJHBP, *Bombyx mori* (AF098304_1); GmJHBP, *Galleria mellonella* (AAN06604.3); MsJHBP, *Manduca sexta* (AAA21588.1). AaJHBP, *Aedes aegypti* (AAL60239.1, Diptera); DmJHBP, *Drosophila melanogaster* (AAM52608.1, Diptera); PhJHBP, *Pediculus humanus* (XP_002432614.1, Phthiraptera); CqJHBP, *Culex quinquefasciatus* (XP_001849011.1, Diptera) and ApJHBP, *Acyrthosiphon pisum* (ACH41922.1, Homoptera). The gray line denotes the ligand binding fragment identified in MsJHBP (Touhara and Prestwich, 1992**)**. Triangles below cysteine (Cys) residues denote conserved disulphide bonds, as suggested for HvJHBP (Wojtasek and Prestwich, 1995). Conservation across species is shown by shading; pink for eight to nine species (80-90%), blue for five to seven species (50 or 70%). The blue lines indicate the fragment JHB. Dots indicate artificial gaps introduced to maximize nucleotide matching. Alignments were performed using DNAMAN (ver. 8.0, Lynnon Biosoft).

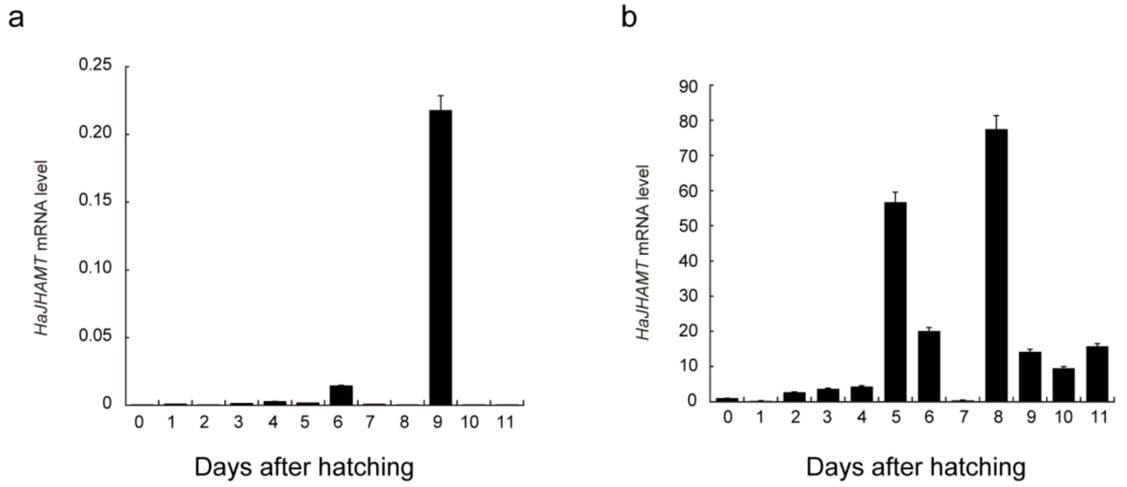

Figure S5. Transcription of (a) *HaJHAMT* and (b) *HaJHBP* in whole *H. armigera* larvae of different ages. The correspondence between days after hatching and instars is 0-1 days for 1st instar, 2-4 days for 2nd instar, 5-7 days for 3rd instar, 8-10 days for 4th instar, and 11 days for 5th instar. Bars represent mean ± SE (n = 3 independent qPCRs) of mRNA quantity relative to *actin.*

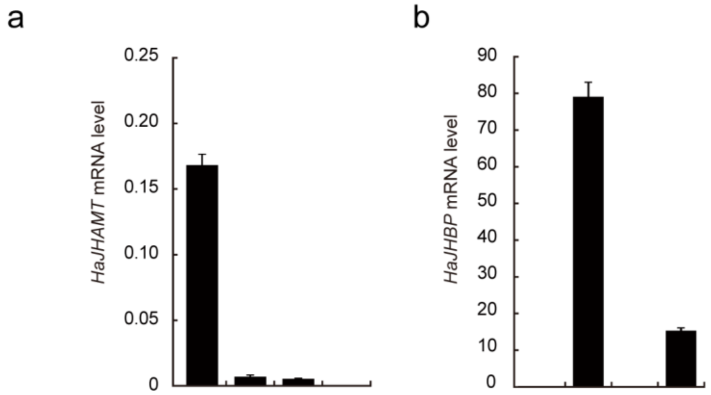

Figure S6. Transcription of (a) *HaJHAMT* and (b) *HaJHBP* in different tissues of fourth instars of *H. armigera*. Head (H), fatbody (F), midgut (M) and epidermis (E). Bars represent mean ± SE (n = 3 independent qPCRs) of mRNA quantity relative to *actin.*

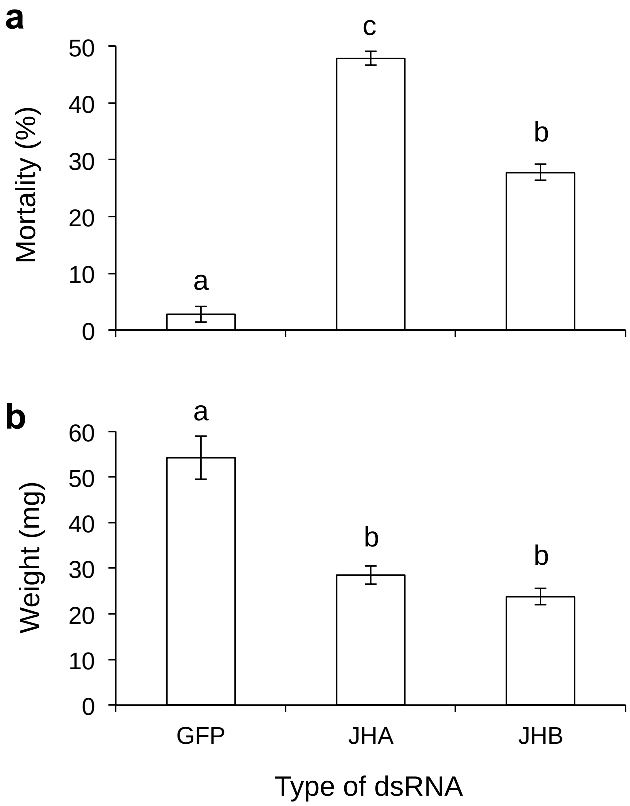

Figure S7. Efficacy of JHA and JHB dsRNA in artificial diet against *H. armigera* larvae. (**a**) Mortality (%). (**b**) Weight. We included dsRNA from GFP as a control. Bars show means and SE based on three replicates of 23 to 24 larvae. Different letters above bars indicate significant differences between treatments ($P < 0.01$, Tukey's HSD). See **Data S1** for details.

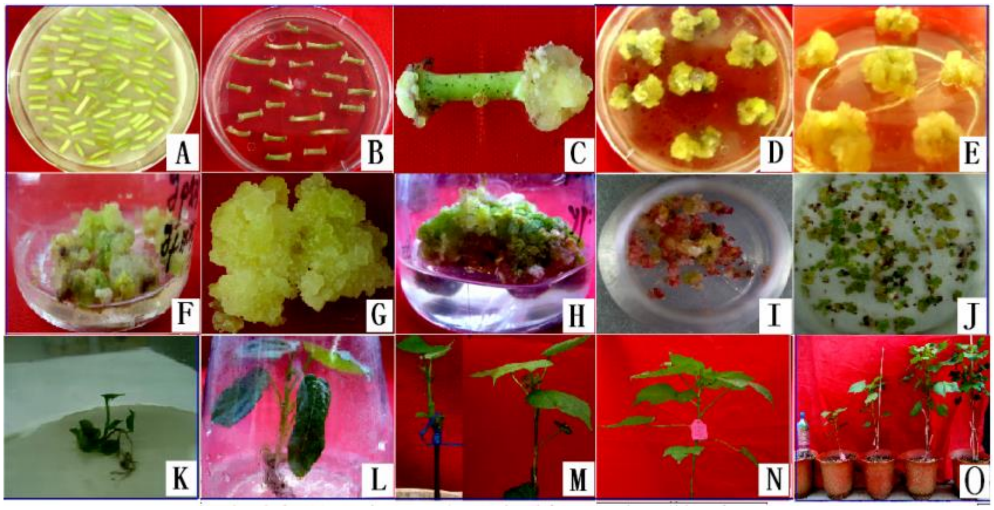

Figure S8. Development of transgenic cotton by *Agrobacterium*-mediated transformation. A: Hypocotyl infections with *Agrobacterium*. B-D: Hypocotyl and hypocotyl-derived calli. E-H: Calli resistant to kanamycin. I-J: Embryonic calli induction. K-L: *In vitro* transgenic cotton plantlet. M-O: Grafted seedling production.

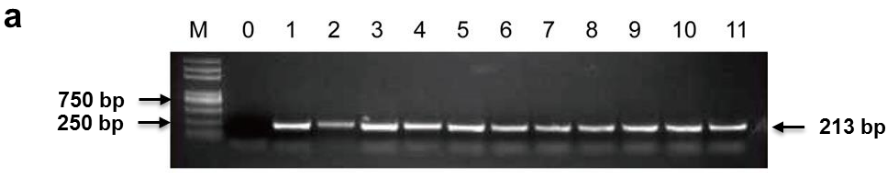

GCCAACACTTGTCACTACTTTCTCTTATGGTGTTCAATGCTTTTCAAGATACCCAGATCATATGAAGCGGC
ACGACTTCTTCAAGAGCGCCATGCCTGAGGGATACGTGCAGGAGAGGACCATCTTCTTCAAGGACGAC
GGGAACTACAAGACACGTGCTGAAGTCAAGTTTGAGGGAGACACCCTCGTCAACAGGATCGAGCTTAA
GGGAAT

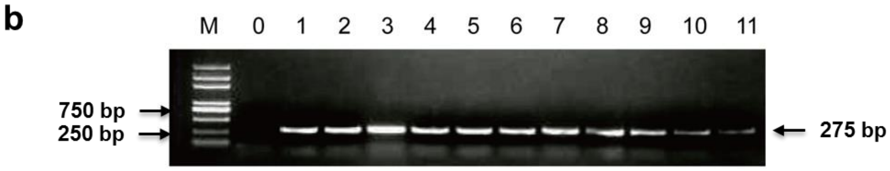

ACATTCTCCATCCTCGCTTAGAAGGTTGTATATGTTGTTGTATGCTCGTTCTTGGTTATTAACCCAGTGCA
GAGCGTAGAACGAGAATACGTGGTCGAAGTTTCCCTTCATGCCTTCAGGTAGATCTCCCTCGATATCAAG
CACGGTGAAAGAAGTCTGTTCGTTACAATGATGATCATTCGCGAAGTTCACCATCTTCTCGCTAATATCAC
AGCCAAGCAACTTATACTCAGTAGGGATATATTTCTTCAGCATGTTAGTCACGCTTCCATCCC

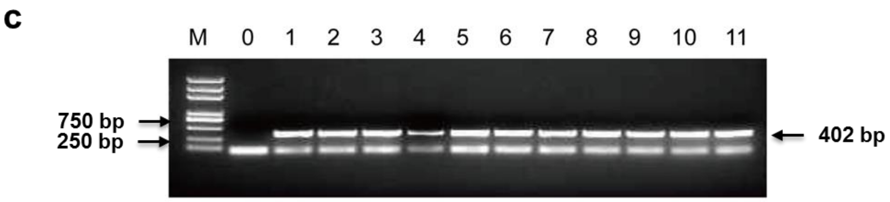

GGCTTGGGACTGGTCTATGATTTCAAAAACATAAATATAACTGGACTGAAGACGCAGCAGATATCAGACTT
CAAAATGGATACAGACAAGAAATCTGTGGTTTTAAAAACAAAAGCTGTTTTGAATATCGTGGGTGATGTCA
AAATCGAATTCGCCAAGCAAAATAAAGTTTTTAATGGAGCTTATACAGCTTCAACAACTGCTATAGGAAGC
TCACAATATGGCTACAGTTTTAAGAAAAAAGATGATAAAGATCACTTCGTAGTCGGCCCAGAGGTGAATAC
ATGTGAAATCATTGGAGAACCGAACGTGGATATTGGAGATGATTTACAAAAGGCCTTGGACAGTGACTCC
GATGCACAGGCTCTGAAACCTGATTACGAGACTAACAAGGTGGCTTT

Figure S9. PCR detection of transgenic T_3 cotton plants. (**a**) GFP transgenic cotton. (**b**) JHA transgenic cotton. (**c**) JHB transgenic cotton. M, Molecular size marker. 0, Non-transgenic plants as negative control. 1, Binary plasmid used for transformation as positive control. 2-11, Transgenic plants (*n* = 10 plants for each type of transgenic cotton). Arrows on the right indicate the expected size of PCR products. The consensus sequence based on sequencing four PCR positive amplicons for each transgene is provided below each gel image. In all cases, the consensus sequence matched the transgene sequence.

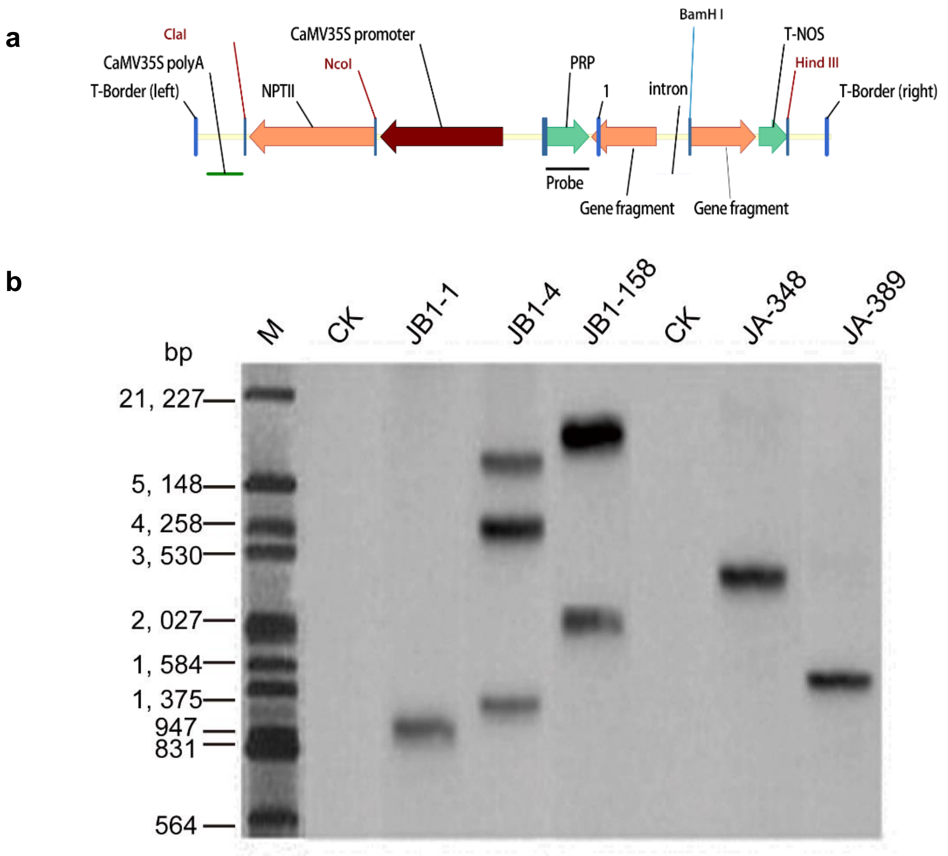

Figure S10. Southern blot detection of transgenic T_3 plants. (**a**) Positions of the probe and the restriction sites in the transgenic vector. One part of the sequence of the binary vector was used as a probe (see Figure S8). (**b**) Southern blot analysis of transgenic cotton. Genomic DNA was digested with Hind III and probed with a DIG-11-dUTP-labelled DNA fragment. M, Molecular size marker, CK, non-transgenic cotton as negative control. We tested five lines of transgenic cotton (from left to right): JB1-1, JB1-4, JB1-158, JA-348 and JA-389. The JA and JB lines were transformed to produce dsRNA from JHA and JHB, respectively. The number of bands indicates the number of integration sites per line: one for JB1-1, JA-348 and JA-389; two for JB1-158; and three for JB1-4. We used lines JA-389 and JB1-1 subsequently and refer to them as JHA and JHB cotton, respectively.

TAGTGGATCCCACATGTTTGAATTTGAAACTTAGTACGCAAGTACTTATAGTGTGCGGGA
GCGTTATTTAGCTTTGAGGGAGCAATCTCGTAAATCGGGGGCCCACAAAAAAAAAAGCG
CGGCCATCCGGTAATATTATACGGATGGCCGCTTTTTGGAGCGTGAGGATTTTGAAATGA
TTTCTCAAATTACGATAATGCCATTTGGGGTACACCTATATATTGCACCCCGTTACACCGAT
TGCCAGAGAATTAGAGTGTACACCGATTGCCACCAT

Figure S11. Probe sequence for Southern analysis of transgenic cotton that matches a portion of the binary vector sequence.

Table S1. Primers used in this study.

Name	Sequence (5'-3')	Purpose
P1-F	GAAAATAAATAACAAGAATCATAAAC	Full length of *HaJHAMT*
P1-R	AAAACTAAACCTGAAGAGATACACTA	
P2-F	ATHGAYCCHHTDRYACCCTC	Middle portion of *HaJHBP*
P2-R	GTGBAYRRMMNYDRYRTARVMNRBY	
P3-F	CACCTCTGGGCCGACTACGAAGTAATCT	*HaJHBP* 5'RACE
P3-R	CTAATACGACTCACTATAGGGCAAGC	
P4-F	5'-(T)25VN-3'	*HaJHBP* 3'RACE
P4-R	ACCACGACTTTTAACTCAGGAAT	
P11-R	GACGAAGGCTTGGGACTGGT	
P12-F	CCTGGTATTGCTGACCGTATGC	*Actin* qRT-PCR
P12-R	CTGTTGGAAGGTGGAGAGGGAA	
P13-F	GGGTACTCGCGCGCAACAACAA	*HaJHAMT* qRT-PCR
P13-R	ATCCTGCGAGTCAGGTTTTCG	
P14-F	ATTGCTTGCATTTGCGAGTTGTGT	*HaJHBP* qRT-PCR
P14-R	TCCGGAAAACCATTGCTAGT	
P15-F	GCCAACACTTGTCACTACTTTCT	*GFP* PCR of cotton
P15-R	ATTCCCTTAAGCTCGATCCTG	
P16-F	ACATTCTCCATCCTCGCTTAG	*JHA* PCR of cotton
P16-R	GGGATGGAAGCGTGACTAAC	
P17-F	GGCTTGGGACTGGTCTATGAT	*JHB* PCR of cotton
P17-R	TAAAGCCACCTTGTTAGTCTCGT	

Table S2 Two-way ANOVA: Effects of year (2015 vs. 2016) and type of pyramid (Bt + JHA vs. Bt + JHB) on mortality and development time of resistant strain SCD-r1 of *H. armigera* in cotton leaf bioassays.

A. Mortality (%)

Source	SS	df	MS	F	P	
Year	33.33	1	33.33	2.94	0.12	
Pyramid	161.33	1	161.33	14.23	0.0054	**
Year X pyramid	5.34	1	5.34	0.47	0.51	
Error	90.67	8	11.33			
Total	290.67	11				

B. Development time (days from neonate to pupation)

Source	SS	df	MS	F	P
Year	0.05	1	0.05	0.91	0.37
Pyramid	0.05	1	0.05	0.91	0.37
Year X pyramid	0.03	1	0.03	0.55	0.48
Error	0.44	8	0.06		
Total	0.57	11			

**The only significant effect was the higher mean mortality on Bt + JHB cotton (74.3%, SE = 1.4%) than on Bt + JHA cotton (67.3%, SE = 1.5%) (P = 0.0054). All other main effects and interactions were not significant.

Table S3. Parameter values used in simulations. See **Tables S4** and **S5** for fitness values used to simulate a pyramid of Bt + JH cotton.

Parameter	Value(s)	Source
Fitness on Bt cotton (h = 0.5)		
s_1s_1	0.22	**Figure 3**
r_1s_1	0.61	See text
r_1r_1	1.00	**Figure 3**
Fitness on JH cotton (h = 0.5, **Figure 5a**)		
s_2s_2	0.32	**Figure 3**
r_2s_2	0.66	See text
r_2r_2	1.00	See text
Fitness on JH cotton (h = 0.2, **Figure 5b**)		
s_2s_2	0.32	**Figure 3**
r_2s_2	0.456	See text
r_2r_2	1.00	See text
Fitness on refuge plants (with minor, additive cost, **Figure 5b**)		
$s_1s_1$and s_2s_2	1.00	By definition
r_1s_1 and r_2s_2	0.95	**Figures 3 and 4**
$r_1r_1$and r_2r_2	0.90	**Figures 3 and 4**
Initial resistance allele frequency		
Bt cotton	0.05	Jin *et al.* 2015
JH cotton	0.001, 0.01	Carrière et al. 2010
Generations per year on cotton	3	Wu and Guo 2005
Effective refuge (%)[a]	5, 10, 25, 50	Jin *et al.* 2015

[a]Effective refuge percentage for the three generations during which *H. armigera* fed on cotton each year. This parameter was estimated as 56% for northern China (Jin *et al.* 2015).

Table S4. Fitness of the nine *H. armigera* genotypes on pyramided Bt + RNAi cotton in simulations as a function of dominance of resistance to the pyramid (h_p). **(a)** Realistic scenario. **(b)** Optimistic scenario. The redundant killing factor (RKF) is calculated as 1 – (fitness on the pyramid of r_1r_1 s_2s_2 – s_1s_1 s_2s_2) and varies from 0 for no redundant killing to 1 for complete redundant killing (Brévault*et al.* 2013).In all simulations of the pyramid, RKF was 0.79 [= 1 – (0.30 – 0.09)].

Dominance of resistance to pyramid (h_p)	Genotype-specific fitness on pyramided Bt + JH cotton								
	r_1r_1 r_2r_2	r_1r_1 r_2s_2	r_1r_1 s_2s_2	r_1s_1 r_2r_2	r_1s_1 r_2s_2	r_1s_1 s_2s_2	s_1s_1 r_2r_2	s_1s_1 r_2s_2	s_1s_1 s_2s_2
**a**) 0.25	1.00	0.65	0.30	0.65	0.318	0.195	0.30	0.195	0.09
**b**) 0.10	1.00	0.44	0.30	0.65	0.181	0.150	0.30	0.132	0.09

Table S5. Fitness of the nine *H. armigera* genotypes on refuge plants in simulations of pyramided RNAi + Bt cotton with a minor, additive fitness cost (optimistic scenario, **Figure 5b)**. Each resistance allele decreases fitness by 0.05. With no fitness cost, all genotypes have fitness = 1 on refuge plants (realistic scenario, **Figure 5a)**.

Genotype-specific fitness on refuge plants								
r_1r_1 r_2r_2	r_1r_1 r_2s_2	r_1r_1 s_2s_2	r_1s_1 r_2r_2	r_1s_1 r_2s_2	r_1s_1 s_2s_2	s_1s_1 r_2r_2	s_1s_1 r_2s_2	s_1s_1 s_2s_2
0.80	0.85	0.90	0.85	0.90	0.95	0.90	0.95	1.00

Data S1. Methods

Efficacy of JHA and JHB dsRNA in artificial diet against *H. armigera* larvae. For artificial diet bioassays, we sprayed ca. 3 µg of transgenic bacteria (OD_{600} = 1.0) producing dsRNA of JHA, JHB, or GFP (control) per g of artificial diet (wet weight). Second instars of *H. armigera* from the susceptible 96S strain were starved for 24 h, then put on the treated diet. The diet was changed daily to maintain a consistent bacterial concentration. After 7 days on the diet, we recorded survival and weight of the survivors.

To generate the transgenic bacteria producing dsRNA from JHA, JHB, or GFP, we constructed recombinant L4440 plasmids of *E. coli* strain HT115 (DE3), which is RNase III-deficient and has a T7 RNA polymerase inducible by isopropyl-β-d-thiogalactoside (IPTG) (Timmons and Fire 1998, Timmons et al. 2001). We used previously described methods (Timmons and Fire 1998, Timmons et al. 2001) to construct recombinant L4440 *E. coli* plasmids in which the target DNA fragment (JHA, JHB, or GFP as a control) was transcribed in both directions by the T7 RNA polymerase to yield dsRNA of JHA, JHB, or GFP. We used the standard $CaCl_2$ method to transform the bacterial hosts with recombinant L4440 plasmids (Sambrook and Russell, 2001; Timmons et al., 2001). Competent bacteria were prepared using the standard $CaCl_2$ method and transformed with recombinant L4440 plasmids by the heat shock method (Timmons et al., 2001). Positive colonies confirmed by PCR and sequencing were cultured overnight in 2×YT media containing the appropriate antibiotic. The bacterial cultures were diluted to 1:500 in 2×YT media with antibiotics and grown until the samples reached an optical density of 0.4 at OD_{600}. T7 polymerase was induced by the addition of 0.4 mM IPTG and the cells were cultured for an additional 4 h to OD_{600} 1.0 and collected at 8,000 g.

The *E. coli* expressing dsRNA were killed by exposing them to 80°C for 20 minutes and stored at -20°C until use in diet bioassays as described above.

Computer simulations. To evaluate evolution of resistance by *H. armigera* in northern China to Bt cotton, a sequence of Bt cotton followed by RNAi cotton, and a pyramid of Bt + RNAi cotton, we used a previously described, deterministic, two-locus population genetic model (Brévault *et al.*, 2013) with some modifications. As in previous work (Brévault *et al.*, 2013; Jin *et al.,* 2015), we assumed mating occurred randomly between adults of different genotypes. Because of the close proximity of small plantings of Bt cotton and non-transgenic host plants in northern China as well as the extensive dispersal and gene flow of *H. armigera* in northern China (Wu and Guo, 2004; Lu *et al.*, 2012) and globally (Behere *et al.*, 2007; Anderson *et al.,* 2016), we also assumed that mating occurred at random among adults emerging from transgenic cotton and non-transgenic host plants (Jin *et al.,* 2015).

Initial frequency of resistance. We set the initial resistance allele frequency as 0.05 for Bt cotton (r_1) based on 2013 Cry1Ac bioassay data for *H. armigera* in northern China, which includes the increase in this frequency that occurred since Bt cotton was first grown widely in northern China in 1997 (Jin *et al.,* 2015). Lacking data on the initial frequency of alleles conferring resistance to RNAi cotton (r_2), we set this to 0.001, which is a standard estimate for populations that have not been exposed previously to a particular toxin or control method (Carrière *et al.*, 2010).

Dominance. Dominance of resistance (h) can be calculated for a single resistance locus as:

(i) $h = (W_{rs} - W_{ss}) / (W_{rr} - W_{ss})$

where W_{ss}, W_{rs}, W_{rr} are the fitnesses of *ss*, *rs*, and *rr,* respectively (Liu and Tabashnik, 1997). Values of *h* vary from 0 for completely recessive resistance to 1 for completely dominant resistance.

For simulations with Bt cotton, we set *h* = 0.5, indicating additive inheritance of resistance. This assumption is a reasonable simplification based on data showing that resistance of *H. armigera* in northern China to Cry1Ac and Bt cotton can be affected by different alleles that are recessive, additive or dominant (Jin *et al.,* 2015). Previous simulations show that results with a single resistance allele with additive effects (*h* = 0.5) are similar to those from more complex modelling incorporating three different alleles that have different levels of dominance (*h* = 0, 0.5 and 1) (Jin *et al.,* 2015).

For simulations with RNAi cotton, we used two values for dominance of resistance: *h* = (a) 0.5 or (b) 0.2. We consider 0.5 a realistic estimate because of the substantial survival of susceptible (SCD) larvae on RNAi cotton leaves in the 2015 and 2016 bioassays (Figure 4). We simulated responses to JHB cotton, which had numerically higher efficacy than JHA cotton (Figure 4). On JHB cotton, mean survival of susceptible larvae was 27.2% (Figure 4). Resistance is expected to be completely recessive (*h* = 0) if the survival of susceptible (*ss*) larvae on transgenic plants is <0.01% (Tabashnik *et al.*, 2013; USEPA, 1998). As survival of susceptible larvae increases, survival of heterozygous larvae (*rs*) also increases, yielding higher values of *h*. Thus, given the 27.2% survival of susceptible larvae on JHB cotton, we used *h* = 0.5 and *h* = 0.2 as realistic and optimistic estimates for resistance to JHB cotton.

For simulations with a pyramid of Bt + RNAi cotton, we used the parameter h_p to describe dominance of resistance to the pyramid (Brévault *et al.*, 2013) as follows:

(ii) $h_p = (W_{r1s1r2s2} - W_{s1s1s2s2}) / (W_{r1r1r2r2} - W_{s1s1s2s2})$.

Values of h_p vary from 0 for completely recessive resistance to 1 for completely dominant resistance. We rearranged equation (ii) to solve for the fitness of double heterozygotes:

(iii) $W_{r1s1r2s2} = h_p \ (W_{r1r1r2r2} - W_{s1s1s2s2}) + W_{s1s1s2s2.}$

To model the pyramid, we set $h_p = 0.25$ in realistic simulations (a) and $h_p = 0.10$ in optimistic simulations (b). These values of h_p are the product of the dominance of resistance for each of the two components of the pyramid (h_1 for Bt cotton and h_2 for RNAi cotton): (a) 0.5 X 0.5 and (b) 0.5 X 0.2.

Fitness on transgenic cotton and non-transgenic refuge plants. We based genotypic-specific fitness values (Tables S3 and S4) on the 2015 and 2016 data from bioassays conducted on transgenic and non-transgenic cotton (Figure 3). In all simulations, we defined the fitness of susceptible homozygotes (s_1s_1 , s_2s_2, and $s_1s_1s_2s_2$) on non-transgenic cotton (W0) as 1. We assumed that fitness on each type of transgenic cotton relative to non-transgenic cotton is proportional to survival on the transgenic cotton relative to non-transgenic cotton. We set the fitness to 1 for r_1r_1 on Bt cotton, because for the Bt-resistant strain (SCD-r1, putative genotype r_1r_1), the bioassay data reported here reveal no significant difference in survival or development time between Bt cotton (means = 83% survival and 21 days, respectively) and non-transgenic cotton (means = 85% survival and 21 days, respectively) (Figure 4). Although data on resistance to RNAi cotton are not available, we also set fitness of r_2r_2 on RNAi cotton as 1, because survival of susceptible larvae was higher on RNAi cotton than on Bt cotton (Figure 4). Assuming fitness of 1 for r_1r_1 on Bt cotton and r_2r_2 on RNAi cotton is conservative, because resistance would evolve slower if the fitness was <1 in either case (i.e., incomplete resistance (Carrière *et al.*, 2010).

Data S2. References

Anderson, C.J., Tay, W.T., McGaughran, Gordon, K. and Walsh, T. K. (2016) Population structure and gene flow in the global pest, *Helicoverpa armigera. Mol. Ecol.* **25**, 5296-5311.

Behere, G.T., Tay, W.T., Russell, D.A., Heckel, D.G., Appleton, B.R., Kranthi, K.R. and Batterham, P. (2007) Mitochondrial DNA analysis of field populations of *Helicoverpa armigera* (Lepidoptera: Noctuidae) and of its relationship to *H. zea*. *BMC Evol. Biol.* **7**, 117.

Brévault, T., Heuberger, S., Zhang, M., Ellers-Kirk, C., Ni, X., Masson, L., Li, X. *et al.* (2013) Potential shortfall of pyramided transgenic cotton for insect resistance management. *Proc. Natl. Acad. Sci. USA,* **110**, 5806–5811.

Carrière, Y., Crowder, D.W. and Tabashnik, B.E. (2010) Evolutionary ecology of insect adaptation to Bt crops. *Evol. Appl.* **3**, 561–573.

Jin, L. Zhang, H., Lu, Y., Yang, Y., Wu, K., Tabashnik, B.E., Wu, Y. *et al.* (2015) Large-scale test of the natural refuge strategy for delaying insect resistance to transgenic Bt crops. *Nat. Biotechnol.* **33**, 169–174.

Kagan, R.M. and Clarke, S. (1994) Widespread occurrence of three sequence motifs in diverse S-adenosylmethionine-dependent methyltransferases suggests a common structure for these enzymes. *Arch. Biochem. Biophys.* **310**, 417–427.

Liu, Y. and Tabashnik, B.E. (1997) Inheritance of resistance to the *Bacillus thuringiensis* toxin Cry1C in the diamondback moth. *Appl. Environ. Microbiol.* **63**, 2218–2223.

Lu, Y., Wu, K., Jiang, Y., Guo, Y. and Desneux, N. (2012) Widespread adoption of Bt cotton and insecticide decrease promotes biocontrol services. *Nature,* **487,** 362–365.

Sambrook, J. and Russell, D.W. (2001) Molecular Cloning. 3rd edn. Cold Spring Harbor, New York.

Timmons, L., and Fire, A. (1998) Specific interference by ingested dsRNA. *Nature* **398**, 854.

Timmons, L., Court, D.L., and Fire, A. (2001) Ingestion of bacterially expressed dsRNAs can produce specific and potent interference in *Caenorhabditis elegans. Gene* **263**, 103-112.

Touhara, K. and Prestwich, G.D. (1992) Binding site mapping of a photoaffinity-labeled juvenile hormone binding protein. Biochem. Bioph. Res. Co. **182**, 466–473.

United States Environmental Protection Agency (USEPA). (1998) Final report of the FIFRA Scientific Advisory Panel Subpanel on Bacillus thuringiensis (Bt) plant-pesticides and resistance management. Available at: https://archive.epa.gov/scipoly/sap/meetings/web/pdf/finalfeb.pdf [Accessed September 2, 2016]

Wojtasek, H. and Prestwich, G.D. (1995) Key disulfide bonds in an insect hormone binding protein: cDNA cloning of a juvenile hormone binding protein of *Heliothis virescens* and ligand binding by native and mutant forms. *Biochemistry,* **34**, 5234–5241.

Wu, K.M. and Guo, Y.Y. (2004) The evolution of cotton pest management practices in China. *Annu. Rev. Entomol.* **50**, 31–52.
